# Prognostic values and prospective pathway signaling of MicroRNA-182 in ovarian cancer: a study based on gene expression omnibus (GEO) and bioinformatics analysis

**DOI:** 10.1186/s13048-019-0580-7

**Published:** 2019-11-08

**Authors:** Yaowei Li, Li Li

**Affiliations:** 1 Department of Gynecologic Oncology, Affiliated Tumor Hospital of Guangxi Medical University, Key Laboratory of Early Prevention and Treatment for Regional High Frequency Tumor, Ministry of Education, Nanning, Guangxi China; 2 Department of Gynecology and obstetrics, Shangyu People’s Hospital, Shangyu, Zhejiang, China

**Keywords:** Ovarian cancer, miR-182, Differentially expressed genes, Functional enrichment analysis, Protein-protein interaction, Survival analysis

## Abstract

**Background:**

Ovarian carcinoma (OC) is a common cause of death among women with gynecological cancer. MicroRNAs (miRNAs) are believed to have vital roles in tumorigenesis of OC. Although miRNAs are broadly recognized in OC, the role of has-miR-182-5p (miR-182) in OC is still not fully elucidated.

**Methods:**

We evaluated the significance of miR-182 expression in OC by using analysis of a public dataset from the Gene Expression Omnibus (GEO) database and a literature review. Furthermore, we downloaded three mRNA datasets of OC and normal ovarian tissues (NOTs), GSE14407, GSE18520 and GSE36668, from GEO to identify differentially expressed genes (DEGs). Then the targeted genes of hsa-miR-182-5p (TG_miRNA-182-5p) were predicted using miRWALK3.0. Subsequently, we analyzed the gene overlaps integrated between DEGs in OC and predicted target genes of miR-182 by Gene Ontology (GO), Kyoto Encyclopedia of Genes and Genomes (KEGG) pathway enrichment analysis. STRING and Cytoscape were used to construct a protein-protein interaction (PPI) network and the prognostic effects of the hub genes were analyzed.

**Results:**

A common pattern of up-regulation for miR-182 in OC was found in our review of the literature. A total of 268 DEGs, both OC-related and miR-182-related, were identified, of which 133 genes were discovered from the PPI network. A number of DEGs were enriched in extracellular matrix organization, pathways in cancer, focal adhesion, and ECM-receptor interaction. Two hub genes, MCM3 and GINS2, were significantly associated with worse overall survival of patients with OC. Furthermore, we identified covert miR-182-related genes that might participate in OC by network analysis, such as DCN, AKT3, and TIMP2. The expressions of these genes were all down-regulated and negatively correlated with miR-182 in OC.

**Conclusions:**

Our study suggests that miR-182 is essential for the biological progression of OC.

## Background

Ovarian carcinoma (OC) is a common cause of death among women with gynecological cancer [1]. Owing to the lack of specific symptoms and methods for early screening, approximately 75% of women have an advanced stage of the disease at diagnosis, which is consequently associated with poor outcome [2]. Therefore, it is urgent to determine the underlying mechanisms and develop new strategies for OC treatment.

MicroRNAs (miRNAs or miRs), a group of endogenous non-coding RNA molecules, can repress gene expression by mRNA degradation**/**destabilization or through impaired translation [3–5]. The abnormal expression of miRNAs occurs in a variety of tumors and often appears to be associated with altered malignant potential, such as changes in tumor cell development, cell proliferation, and apoptosis [6–8].

Increasing studies have revealed that aberrant expression of has-miR-182-5p (miR-182) contributes to the biological processes of various types of cancer. For example, some studies have indicated that over-expression of miR-182 shows increased tumour cell growth and proliferation, highly aggressive features, and tumor progression through repression of a plethora of targets (e.g., CAMK2N1 [9], RAB27A [10] and activation of Wnt**/**β-catenin signal pathway [11]; Perilli et al. [12] have found that circulating miR-182 can serve as a biomarker for tumor progression. However, some studies have shown different conclusions. For example, one study has shown that over-expression of miR-182 inhibits the epithelial to mesenchymal transition and metastasis via inactivation of Met**/**AKT**/**Snail in non-small cell lung cancer cells [13], Sun et al. [14] have reported that the miR-182 expression in cervical cancer is down-regulated and miR-182 induces cervical cancer cell apoptosis by suppressing DNMT3a expression.

MiR-182-related aggressive growth is mainly mediated by the direct regulation of genes associated with tumor invasion and metastasis [15, 16]. Furthermore, it has been found that miR-182 plays oncogenic roles by directly targeting and negatively regulating PDCD4 in ovarian cancer [17]. Previous studies have shown that miR-182 expression levels are significantly up-regulated in ovarian cancer [15, 18], whereas one study has found that miR-182 is down-regulated [19].

Owing to data published on miR-182 expression in OC are partly conflicting and heterogeneous, our study aims to unveil the role of miR-182 in ovarian cancer through investigation of miRNAs expression and identification of putative molecular targets by bioinformatics analysis and analysis based on GEO and literature reviews.

## Materials and methods

### Selection of GEO dataset

We obtained the microarray profiles of OC from the GEO database (Gene Expression Omnibus, http://www.ncbi.nlm.nih.gov/geo/). The following keywords were used in the GEO database: (ovarian) AND (cancer OR carcinoma OR tumor OR neoplasia OR neoplasm OR malignant OR malignancy) AND (microRNA OR miRNA OR noncoding RNA OR ncRNA OR small RNA). The microarray datasets reporting miR-182 expression between OC and normal ovarian tissues (NOTs) were included in our study.

### Study selection and data extraction for literature review

A full-scale literature search was performed in PubMed and Embase (up to March 31, 2019) by using the following terms: (microRNA OR miRNA OR noncoding RNA OR ncRNA OR small RNA) AND (182 OR 182-5p) AND (ovarian) AND (cancer OR carcinoma OR tumor OR neoplasia OR neoplasm OR malignant OR malignancy). Publications were considered eligible if they met the following criteria: (1) studies examining the expression of miR-182 in OC; and (2) NOTs were used as control group. The studies were considered ineligible based on the following criteria: (1) reviews, non-clinical studies, case reports, meta-analyses, and conference abstracts; and (2) absence of control groups.

### Gene ontology enrichment and target prediction analysis

The gene expression profile of GSE14407, GSE18520, and GSE36668 were obtained from Gene Expression Omnibus (GEO, http://www.ncbi.nlm.nih.gov/geo) database. The array data of GSE14407, GSE18520, and xGSE36668 consisted of 12, 53, and 4 OC and 12, 10, and 4 NOTs samples, respectively. All data were analyzed on the GPL570 Platform Affymetrix Human Genome U133 Plus 2.0 (Affymetrix; Thermo Fisher Scientific, Inc., Waltham, MA, USA).

The Limma package (version 3.36.5) in R**/**Bioconductor was used to identify the differentially expressed genes (DEGs) between OC and NOTs [20]. The adjusted *P* value (adj. P.Value) was applied to correct for the occurrence of false positive results using Benjamini and Hochberg false discovery rate (FDR) method by default [21]. The adj. *P*. Value < 0.05 and |log2(FC)| > 1 were set as the cut-off criterion. The original probe-level data in Series Matrix Files were converted into gene symbol based on the downloaded platform annotation files. The expression values of multiple probes corresponding to the same gene were selected by the minimum adj. P.Value.

The targeted genes of hsa-miR-182-5p (TG_miRNA-182-5p) were predicted using miRWALK3.0 (http://zmf.umm.uni-heidelberg.de/apps/zmf/mirwalk2/miRretsys-self.html) [22]. Subsequently, we analyzed the gene overlaps integrated between DEGs in OC and predicted TG_miRNA-182-5p by bioinformatics software. Gene Ontology (GO) and Kyoto Encyclopedia of Genes and Genomes (KEGG) pathway enrichment analysis were performed for the gene overlaps using DAVID database. FDR < 0.05 was set as the cut-off criterion. Protein-protein interaction (PPI) network was constructed based on the gene overlaps using the Search Tool for the Retrieval of Interacting Genes (STRING, version 11.0, https://string-db.org/) database, which was then visualized by Cytoscape software (version 3.7.1) [23]. And confidence score C ≥ 0.7 was set as the cut-off criterion. Then, the Molecular Complex Detection (MCODE) was performed to screen modules of PPI network with degree cutoff = 2, node score cutoff = 0.2, k-core = 2, and max. Depth = 100 [24].

### Survival analysis

Kaplan–Meier plotter (KM plotter, www.kmplot.com) was capable to assess the effect of 54,675 genes on survival using 18,674 cancer samples, include 5143 breast, 1816 ovarian, 2437 lung, 364 liver, and 1065 gastric cancer patients. Based on the median expression level of a particular gene, the patients with OC were divided into two groups (high vs. low). The overall survival of patients with OC was analyzed using a Kaplan–Meier plot. The hazard ratio (HR) with 95% confidence intervals (CI) and log rank *P* value were calculated and displayed.

### Statistical analysis

All data are displayed as mean ± standard deviation (SD) from each group. Student’s t-test was performed to analyze the differences between two groups. Standardized mean difference (SMD) was applied to evaluate the association between miR-182 levels and OC by RevMan 5.3 software. We pooled SMD across GEO datasets using the Mantel–Haenszel formula (fixed-effect model) or the DerSimonian–Laird formula (random effects model). A random-effect model was adopted when the Q statistic was considered significant (*p* < 0.1 or *I*^2^ > 50%), otherwise, a fixed-effect model was used. The relationship of DEGs expression with miR-182 level was analyzed by Spearman’s rank correlation. A two-sided *P*-value < 0.05 was considered statistically significant.

## Results

### MiR-182 expression in OC based on GEO

**Fig. 1:**
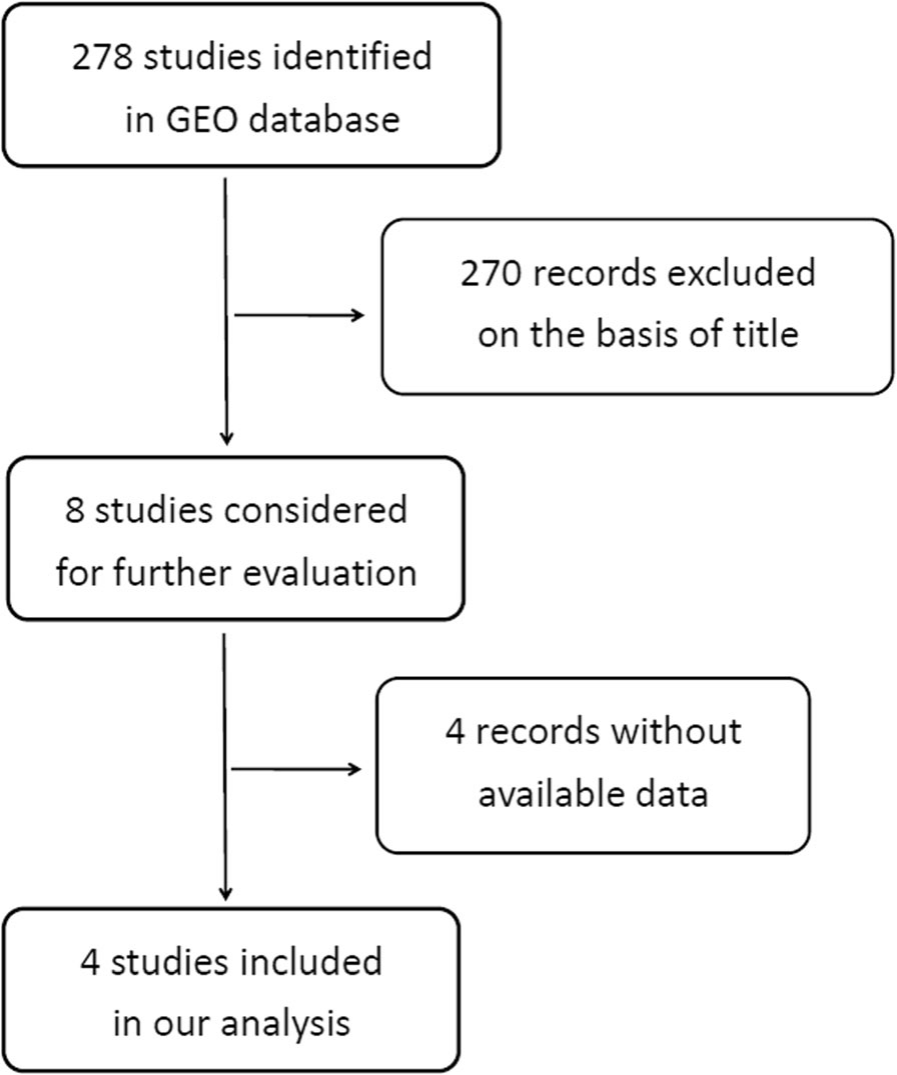
Flow chart of study selection for GEO dataset

**Fig. 2:**
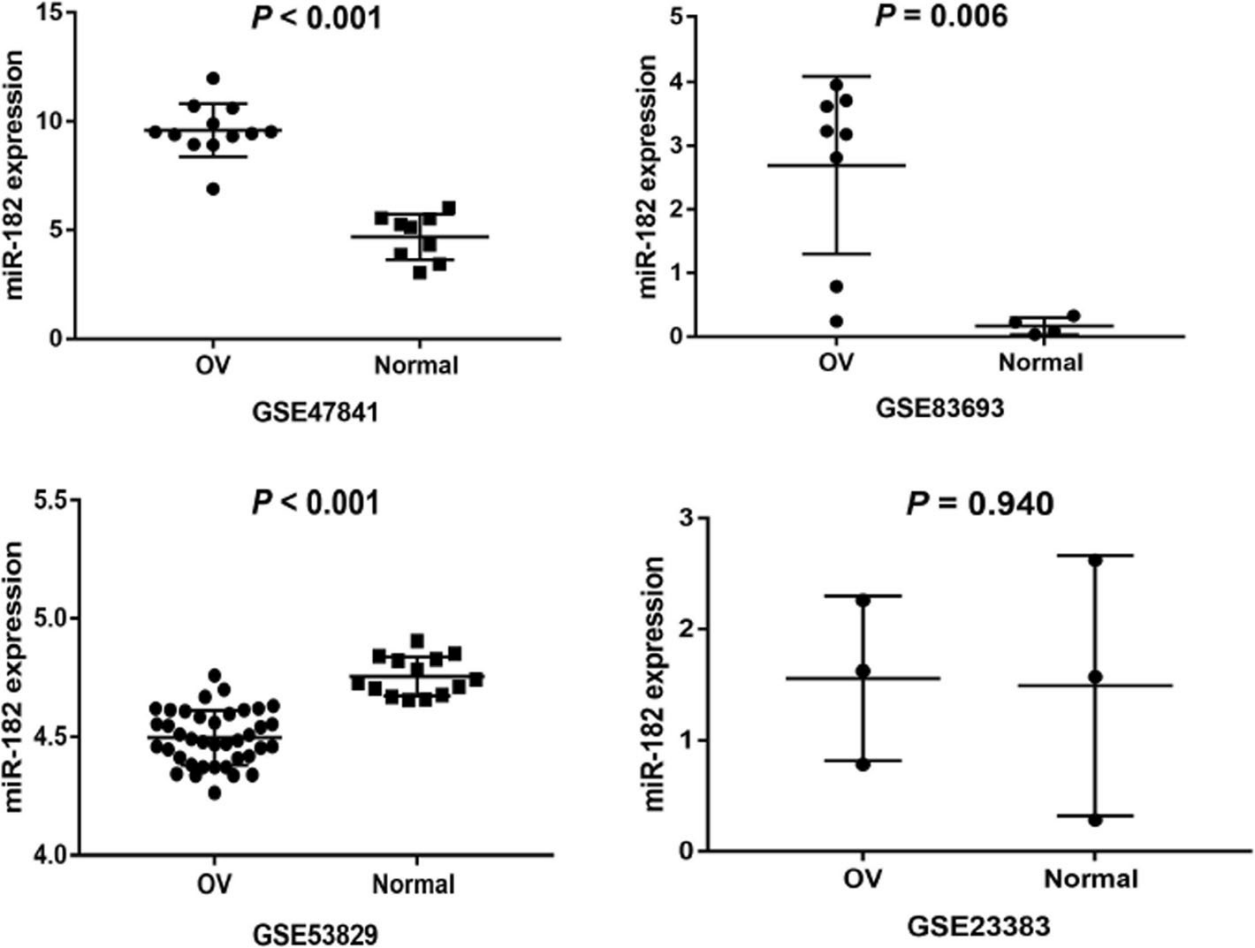
Expression of miR-182 in ovarian cancer and normal ovarian tissues in GEO datasets. OV: ovarian cancer tissue; Normal: normal ovarian tissue; miR-182: hsa-miR-182-5p

**Fig. 3:**
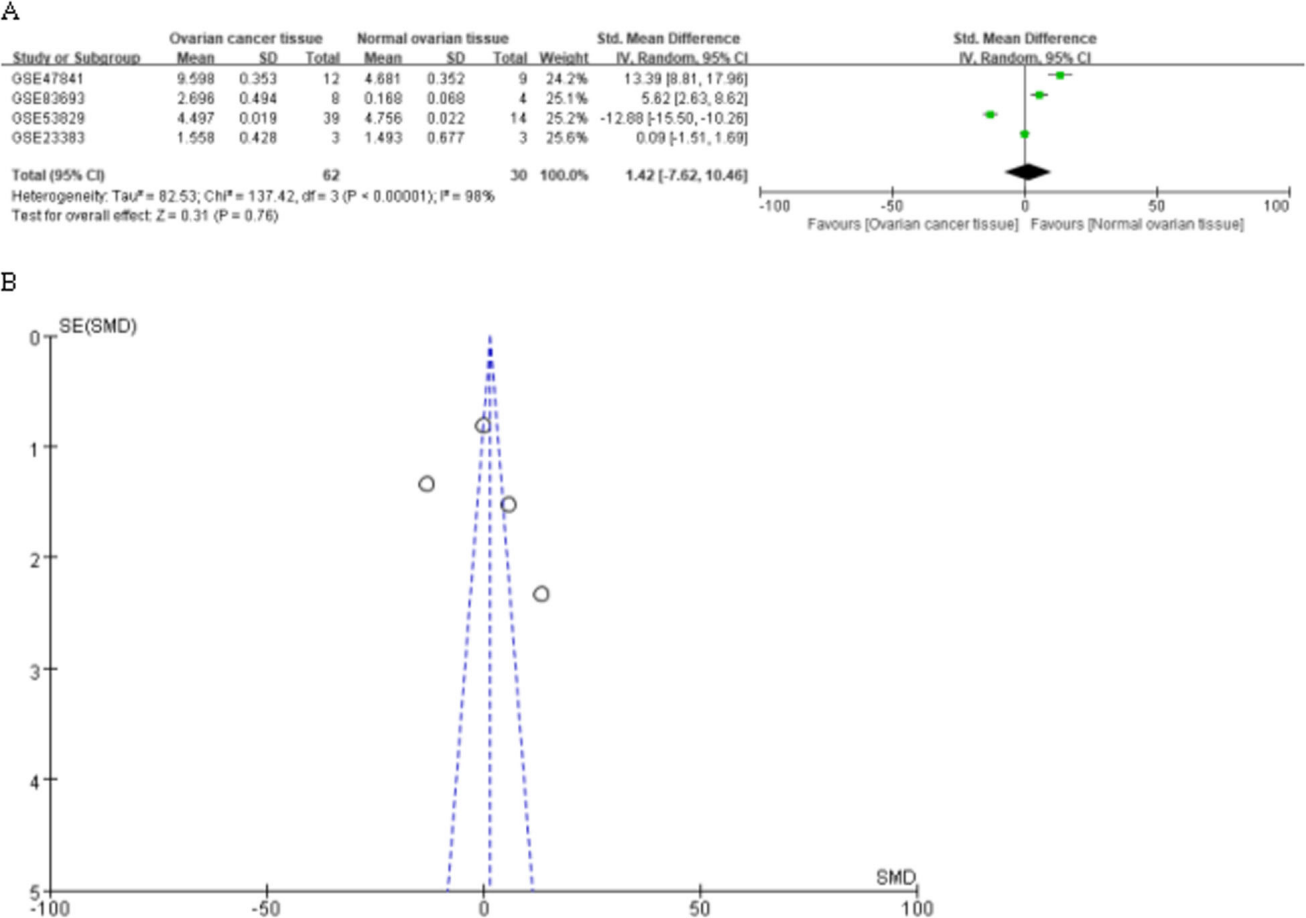
Forest plot (A) and funnel plot (B) of the combined SMD for hsa-miR-182-5p expression between ovarian cancer and normal ovarian tissue by the random effects models

MiR-182 expression was initially assessed in a series of OC and NOTs based on GEO dataset (Fig. 1). A total of four GEO datasets (GSE47841, GSE83693, GSE53829, and GSE23338) were collected in our study. The expression levels of miR-182 in OC tissues were significantly higher than in NOTs in GSE47841 and GSE83693 datasets (*p* < 0.001 and *p* = 0.006; respectively), the expression levels of miR-182 in OC tissues were significantly lower than in NOTs in GSE53829 dataset (*p* < 0.001), whereas no significant difference was found in GSE23338 dataset. Characteristics of studies based on GEO dataset are presented in Table 1 and Fig. 2. However, no significant difference was found between ovarian cancer and normal ovarian tissue groups based on all the included GEO datasets (SMD = 1.42; 95% CI: − 7.62 to 10.46; *p* = 0.76) with significant heterogeneity by random-effected model (*p* < 0.0001, *I*^*2*^ = 98%). The results of forest plot are shown in Fig. 3.

**Table 1:** Characteristics of studies based on GEO dataset

Study	Ovarian cancer tissue			Normal ovarian tissue			t	p
	Mean	SD	n	Mean	SD	n		
GSE47841	9.598	0.353	12	4.681	0.352	9	9.658	< 0.0001
GSE83693	2.696	0.494	8	0.168	0.068	4	3.523	0.006
GSE53829	4.497	0.019	39	4.756	0.022	14	7.689	< 0.0001
GSE23383	1.558	0.428	3	1.493	0.677	3	0.081	0.94
Total	SMD(95%CIs) = 1.42(−7.62,10.46), *p =* 0.76; *I*^2^ = 98%, *p <* 0.00001

### Literature review of miR-182 expression profiles in OC versus NOTs

**Fig. 4:**
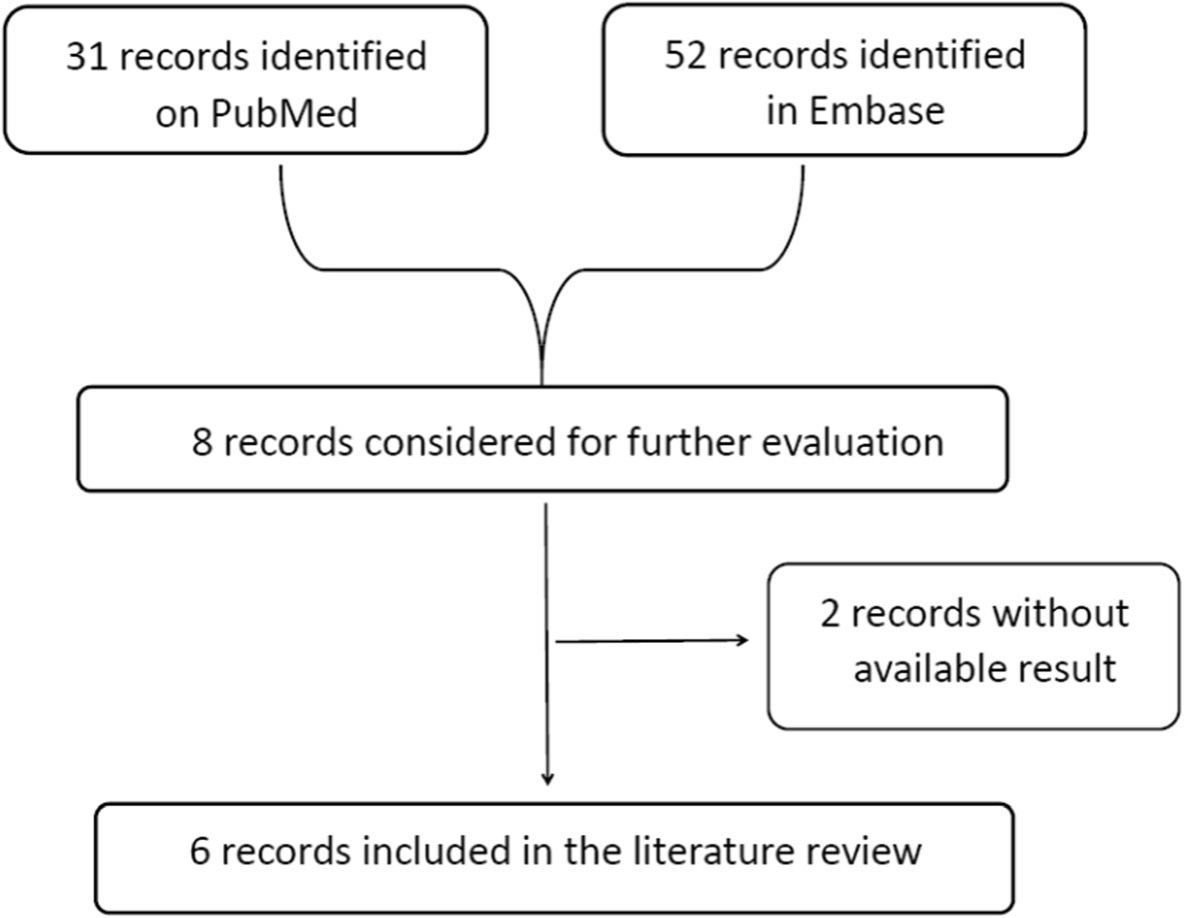
Flow chart of study selection for the literature review

Next, we explored miR-182 expression in OC based on literature data. As shown in Fig. 4, six studies that met the criteria for selection were selected from the literature [15, 17, 19, 25–27]. Five of the six studies included showed that the expression level of miR-182 in OC tissues was significantly higher than that in the NOTs, while one study showed the opposite conclusion (Table 2).

**Table 2:** Overview of the 6 studies selected from literature

Author	Year	Country	Case		Control		Result	Detection methods
			Source name	n	Source name	n		
Wei Lu	2016	China	OC	12	NOTs	8	Down-regulation	qRT-PCR
Barbara Marzec-Kotarska	2016	Poland	EOC	47	NOTs	26	Up-regulation	microRNA microarrays
Lin Wang	2014	China	OC	41	NOTs	15	Up-regulation	Real-time PCR
Bente Vilming Elgaaen	2014	Norway	HGSC	35	NOTs	9	Up-regulation	qRT-PCR
Zhaojian Liu	2012	USA	HG-PSC	56	FT	21	Up-regulation	microRNA microarrays
Yu-Quan Wang	2013	China	OC	13	NOTs	2	Up-regulation	stem-loop RT-PCR

*OC* ovarian cancer, *EOC* epithelial ovarian cancer, *HGSC* High-grade serous ovarian carcinoma; *HG-PSC* high-grade papillaryserous carcinoma; *NOTs* normal ovarian tissues, *FT* fallopian tube tissue

### miR-182 prediction and bioinformatics analyses

#### Data preprocessing and DEGs screening

**Fig. 5:**
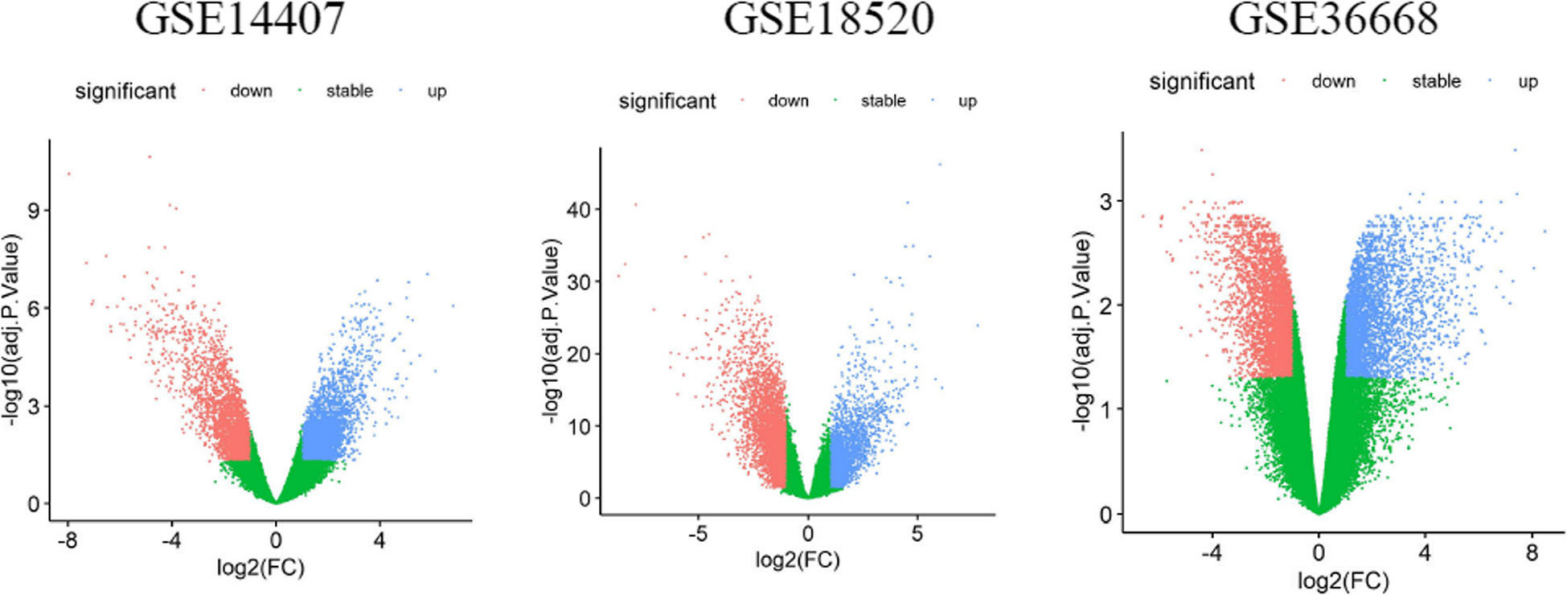
Volcano plot of detectable genome-wide mRNA profiles in ovarian cancer tissue and normal ovarian tissue samples from GSE14407, GSE18520, and GSE36668, respectively. Blue and red plots represent aberrantly expressed mRNAs with P<0.05 and |log(FC)|>1. Blue plots indicate up-regulated genes, red plots indicate down-regulated genes and green plots indicate normally expressed mRNAs. The x-axis is the fold-change value between the expression of mRNAs in ovarian cancer tissues and normal ovarian tissues. The y-axis is the -log10 of the adj.P.Value for each mRNA, representing the strength of the association. adj.P.Value, adjusted P value; FC, fold change

**Fig. 6:**
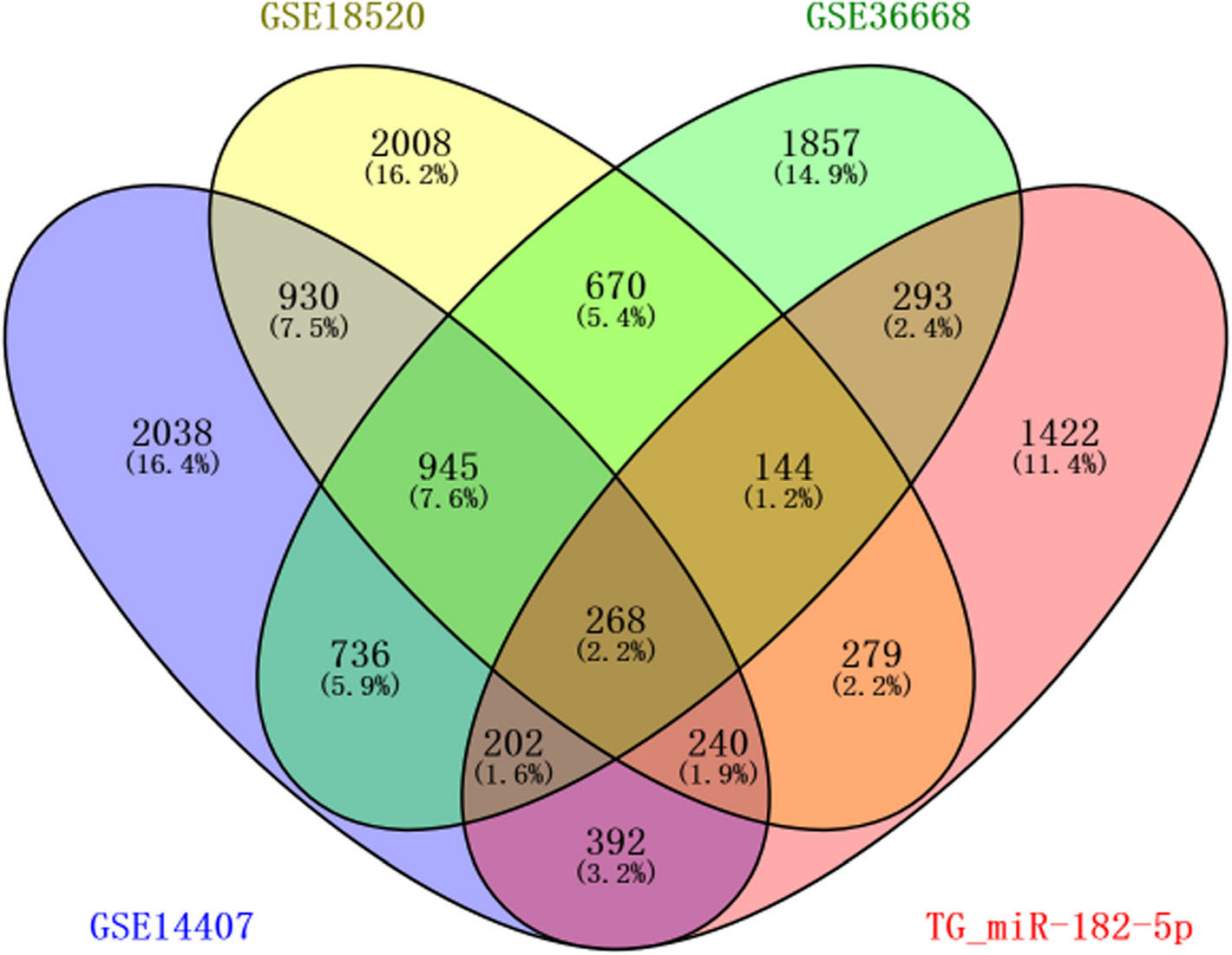
Venn plots of hsa-miR-182-5p-related differentially expressed genes from four datasets ( GSE14407, GSE18520, GSE36668, and TG_miR-182-5p), the overlapping area corresponds to the commonly identified DEGs. DEGs: differentially expressed genes; TG_miR-182-5p, target genes of hsa-miRNA-182-5p.

A total of 5751, 5484, and 5115 DEGs were identified from GSE14407, GSE18520, and GSE36668 datasets, respectively; 1213 common DEGs were screened out in these three datasets with Venny 2.1.0(http://bioinfogp.cnb.csic.es/tools/venny/index.html) [28] (Fig. 5, Fig. 6). Following, based on miRWALK3.0, 3240 predicted TG_hsa-miR-182-5p were obtained, of which 268, were validated in 1213 commonly identified DEGs. There were 130 up-regulated and 138 down-regulated hsa-miR-182-5p-related genes in OC tissues compared with NOTs according to data from Gene Expression Omnibus (Fig. 6, Table 3).

**Table 3:** Top 10 hsa-miR-182-5p-related differentially expressed genes in ovarian cancer tissues compared with normal ovarian tissues according to data from Gene Expression Omnibus (GSE14407)

DEG	logFC	P.Value	adj. P.Value
up-regulated genes			
MECOM	4.902333971	9.81E-08	1.99E-05
TFAP2A	4.745918435	1.88E-08	6.04E-06
DEPDC1	4.032946443	5.66E-09	3.03E-06
AIF1L	3.868411982	3.79E-08	9.54E-06
BIRC5	3.7736042	8.61E-09	3.89E-06
BCL11A	3.534029736	1.11E-06	1.13E-04
L1CAM	3.279255511	1.66E-06	1.49E-04
HMMR	3.262907245	2.33E-06	1.95E-04
NCAPG	3.229344903	1.07E-08	4.36E-06
DTL	3.208682435	1.51E-10	3.76E-07
down-regulated genes			
PDE7B	−5.031930277	6.84E-09	3.37E-06
TCEAL7	−4.928868798	4.70E-09	2.66E-06
DCN	−4.886965887	1.16E-08	4.61E-06
GPM6A	−4.799779935	2.81E-08	7.77E-06
PGR	−4.345763842	2.85E-08	7.83E-06
PPM1E	−4.270922539	1.43E-08	5.13E-06
TMOD2	−4.108261122	5.21E-10	7.49E-07
NKX3–1	−3.851343755	1.34E-07	2.48E-05
TACC1	−3.745549626	5.00E-08	1.18E-05
FGF13	−3.684968538	2.31E-09	1.78E-06

*DEG* differentially expressed gene, *FC* fold-change

#### Functional analysis of miR-182-related DEGs in OC

Functional and pathway enrichment analysis was performed using DAVID. The analysis revealed that numerous target genes were involved in the biological processes, such as nucleus, protein binding, and extracellular matrix organization. Moreover, three KEGG pathways were over-represented in these potential target genes, that is, pathways in cancer, focal adhesion, and ECM-receptor interaction (Table 4).

**Table 4:** Functional and pathway enrichment analysis of hsa-miR-182-5p-related differentially expressed genes in ovarian cancer

Term	Description	Count	*P*-Value	FDR
Cellular components				
GO:0005634	nucleus	120	4.67E-08	6.23E-05
GO:0005737	cytoplasm	108	1.38E-05	1.85E-02
Molecular function				
GO:0005515	protein binding	173	1.58E-07	2.23E-04
GO:0043565	sequence-specific DNA binding	23	1.18E-05	1.67E-02
Biological processes				
GO:0030198	extracellular matrix organization	14	1.00E-05	1.70E-02
KEGG pathway				
hsa05200	Pathways in cancer	22	1.93E-06	2.41E-03
hsa04510	Focal adhesion	15	8.20E-06	1.02E-02
hsa04512	ECM-receptor interaction	10	1.38E-05	1.72E-02

*FDR* false discovery rate, *Count* the number of enriched genes in each term, *GO* gene ontology, *KEGG* Kyoto Encyclopedia of Genes and Genomes

#### PPI network construction and modules selection

**Fig. 7:**
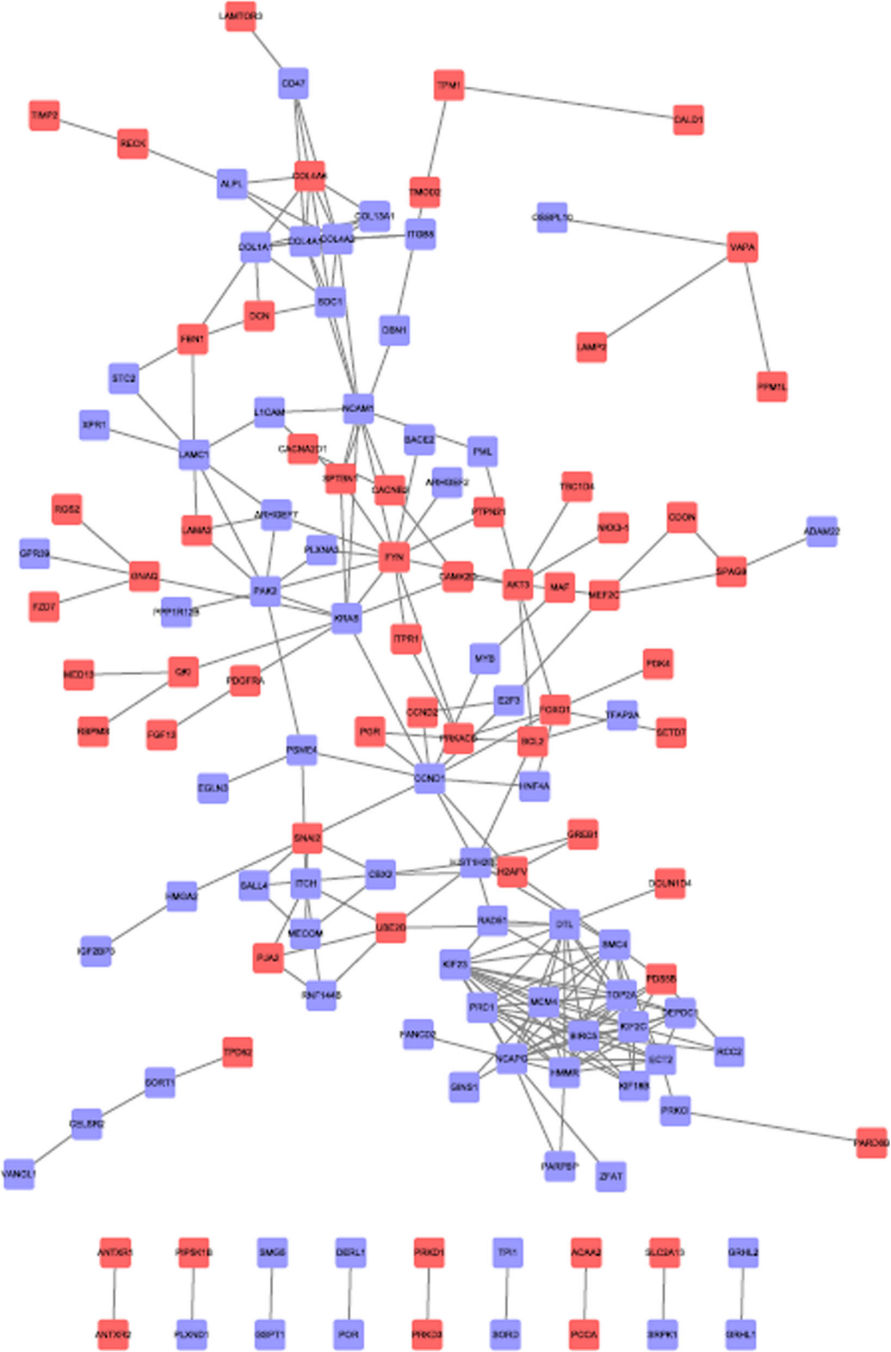
Protein-protein interaction network of hsa-miR-182-5p-related DEGs. Blue nodes stand for up-regulated genes, while red nodes stand for down-regulated genes. The lines represent interaction relationship between nodes. DEGs, differentially expressed genes

**Fig. 8:**
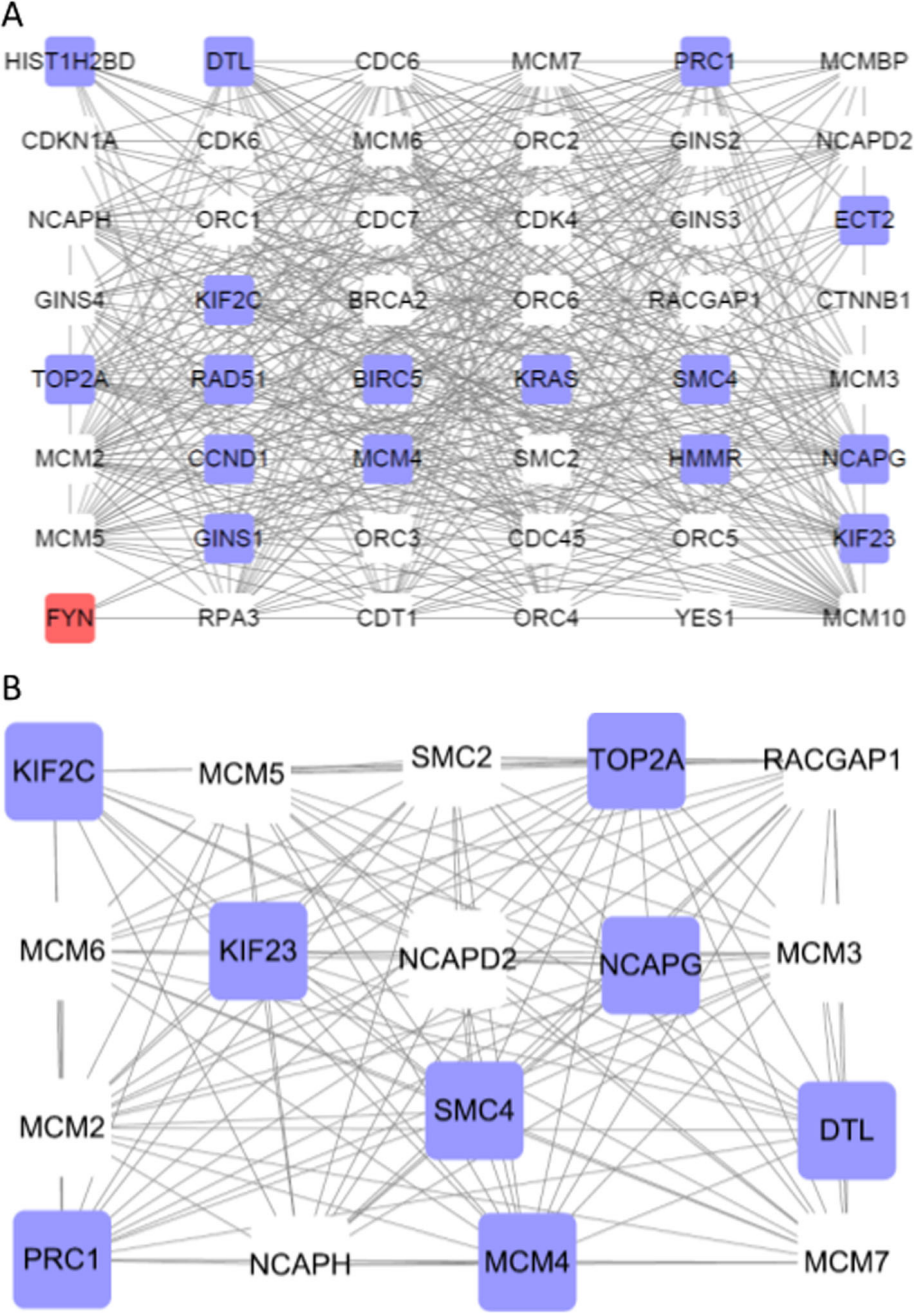
Protein-protein interaction network. (A): Protein-protein interaction network of hube genes of hsa-miR-182-5p-related DEGs. (B): A significant module selected from protein-protein interaction network of hsa-miR-182-5p-related DEGs. Blue nodes stand for up-regulated genes, while red nodes stand for down-regulated genes. The lines represent interaction relationship between nodes. DEGs, differentially expressed genes

The PPI network of miR-182-related DEGs consisted of 153 nodes and 439 edges, including 73 up-regulated genes and 60 down-regulated genes (Fig. 7). Degrees ≥10 was set as the cutoff criterion [29], a total of 28 genes were selected as hub genes, Moreover, there were close correlations among hub genes (Fig. 8a, Additional file 1: Table S1). A significant module was obtained from PPI network of miR-182-related DEGs using MCODE, including 17 nodes and 122 edges (Fig. 8b).

#### Survival analysis

**Fig. 9:**
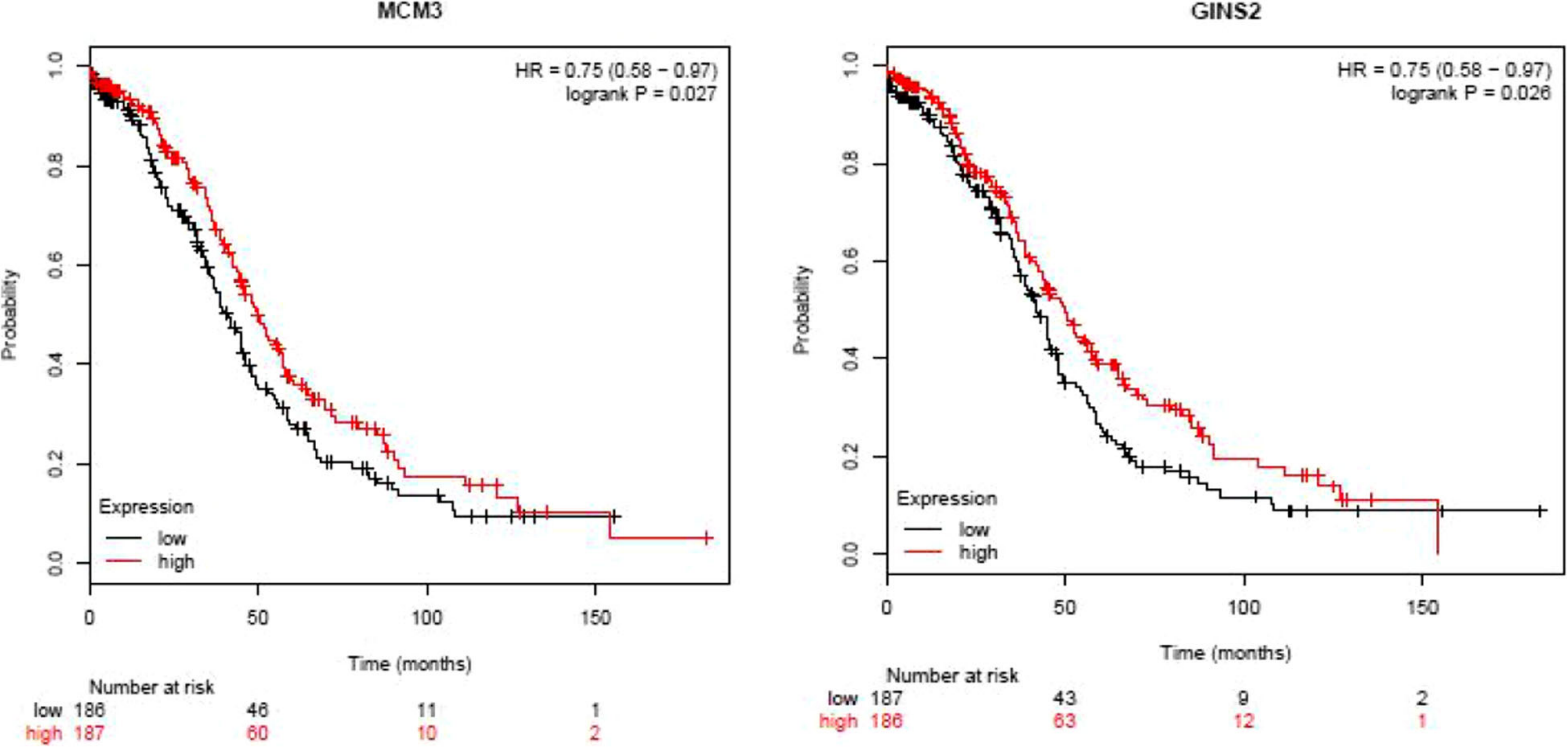
Overall survival analysis of MCM3 and GINS2 expression with prognosis of ovarian cancer patients. The patients with ovarian cancer were divided into two groups (high vs. low), according to the median expression level of MCM3 and GINS2

The prognostic value of 28 hub genes in PPI network was assessed in www.kmplot.com. The overall survival of patients with OC was analyzed depending on the high and low expression of each hub gene. It was found that low mRNA expression of MCM3 (HR 0.75 [0.58–0.97], *P* = 0.027) was associated with worse overall survival for ovarian cancer patients, as well as GINS2 (HR 0.75 [0.58–0.97], *P* = 0.026) (Fig. 9). In the PPI network, a total of 21 genes (TPM1, COL1A1, PDGFRA, UBE2B, MEF2C, SNAI2, CACNA2D1, RECK, FOXO1, FBN1, ANTXR2, NKX3–1, TIMP2, AKT3, RBPMS, EGLN3, DERL1, PRKD1, SLC2A13, MAF, and DCN) were revealed to exert their potential roles in OC by interactions with miR-182 (Table 5, Additional file 2: Figure S1).

**Table 5:** Correlation between hsa-miR-182-5p (miR-182) and target genes in patients with ovarian cancer(*n* = 376)

miRNA	Target gene	r	*p*-value	FDR
hsa-miR-182-5p	TPM1	−0.143	5.61E-03	1.99E-02
hsa-miR-182-5p	COL1A1	−0.154	2.74E-03	1.14E-02
hsa-miR-182-5p	PDGFRA	−0.18	4.40E-04	2.87E-03
hsa-miR-182-5p	UBE2B	−0.149	3.87E-03	1.49E-02
hsa-miR-182-5p	MEF2C	−0.201	8.70E-05	8.01E-04
hsa-miR-182-5p	SNAI2	−0.154	2.84E-03	1.18E-02
hsa-miR-182-5p	CACNA2D1	−0.104	4.44E-02	1.01E-01
hsa-miR-182-5p	RECK	−0.153	2.93E-03	1.21E-02
hsa-miR-182-5p	FOXO1	−0.115	2.57E-02	6.58E-02
hsa-miR-182-5p	FBN1	−0.139	6.81E-03	2.33E-02
hsa-miR-182-5p	ANTXR2	−0.187	2.62E-04	1.90E-03
hsa-miR-182-5p	NKX3–1	−0.136	8.32E-03	2.70E-02
hsa-miR-182-5p	TIMP2	−0.194	1.51E-04	1.23E-03
hsa-miR-182-5p	AKT3	−0.205	6.28E-05	6.32E-04
hsa-miR-182-5p	RBPMS	−0.17	9.57E-04	5.21E-03
hsa-miR-182-5p	EGLN3	−0.113	2.83E-02	7.09E-02
hsa-miR-182-5p	DERL1	−0.164	1.41E-03	6.98E-03
hsa-miR-182-5p	PRKD1	−0.177	5.57E-04	3.44E-03
hsa-miR-182-5p	SLC2A13	−0.115	2.57E-02	6.58E-02
hsa-miR-182-5p	MAF	−0.268	1.26E-07	5.19E-06
hsa-miR-182-5p	DCN	−0.236	3.68E-06	6.45E-05

*miRNA or miR*, microRNA, *FDR* false discovery rate

## Discussion

In current study, we identified aberrantly expressed miR-182 associated with OC through the comparison of miRNA expression profiles in OC tissues with that of NOTs based on data from GEO datasets and published studies. In addition, we identified and analyzed novel markers and potential targets for miR-182 that were involved in the regulation of crucial biological processes in OC by GO analysis, KEGG pathway annotation, protein-protein interaction (PPI) network, and Kaplan-Meier plotter.

To date, there have been only a few studies on the characteristics of miR-182 in OC. In 2012, Liu et al. [15] demonstrated that the expression level of miR-182 was higher in high-grade papillary serous carcinoma (HG-PSC) than in fallopian tube tissues. Subsequently, several other studies also supported the conclusion that miR-182 expression was up-regulated in ovarian cancer tissues [17, 25–27]. Interestingly, a recent study have suggested that the expression level of miR-182 is down-regulated in ovarian cancer tissues compared with paracancerous and NOTs [19]. Based on the data of included GEO datasets, only two sets of GSE datasets (GSE47841 and GSE83693) showed the expression level of miR-182 was up-regulated in OC. Due to the result of the expression level of miR-182 in OC is still controversial, further investigation is necessary to elucidate the role of miR-182 in OC.

MiR-182 is one of the most frequently deregulated miRNA in OC. Marzec-Kotarska et al. [25] demonstrated that miR-182 expression was significantly increased, higher miR-182 expression was linked with significantly shorter overall survival, and Deletion of the PRDM5 locus may play a supportive role in miR-182 overexpression in OC. Additionally, Wang et al. [17] found that in ovarian cancer, miR-182, as an oncogenic miRNA, promoted cell growth, invasion, and chemoresistance by directly and negatively regulating PDCD4. Liu et al. [15] proposed that the oncogenic properties of miR-182 in ovarian cancer may be partly due to its impaired repair of DNA double-strand breaks, negative regulation of breast cancer 1 (BRCA1) and metastasis suppressor 1 (MTSS1) expressions, and positive regulation of oncogene high-mobility group AT-hook 2 (HMGA2). Interestingly, a recent study has found that miR-182 could induce apoptosis of ovarian cancer by regulating of DNTM3a expression [19]. In view of the current situation, it is necessary to further elucidate the molecular mechanism and clinical value that is associated with abnormal expression of miR-182 in OC.

MCM3 is a member of minichromosome maintenance protein family with a critical role in initiation of DNA replication [30]. It is present during cellular proliferation of normal cells, premalignant and neoplastic cells but absent in cells that are in G0 phase [31]. MCM3, presented in a variety of human tumors, is involved in tumor proliferation [32], diagnosis [33], and prognosis. For example, Hua et al. [34] reported that high mRNA expression levels of MCM2 and MCM3 were correlated with a poor outcome and thus might be clinically useful molecular prognostic markers in glioma. Jankowska-Konsur et al. [35] found that MCM3 was reliable parameters for the correlation with clinical stage of mycosis fungoides and MCM3 expression was of prognostic value in mycosis fungoides. In current study, survival analysis of the hub genes related to target genes of miR-182 revealed that MCM3 was highly associated with poor prognosis of patients with OC. GINS complex subunit 2 (GINS2), is a member of the GINS complex, composed of GINS1, GINS2, GINS3, and GINS4, which is involved in DNA replication [36]. GINS2 is up-regulated in a variety of aggressive tumors. For example, Liu et al. [37] found that lung cancer tissues over-expressed GIN2, which was connected to lung cancer metastasis. Recent years, some studies have found that GINS2 is a novel prognostic biomarker and promoted tumor progression in early-stage cervical cancer [38]; GINS2 is closely related to the occurrence and development of glioma, and may become a prognostic marker for glioma patients [39]; GINS2 is markedly expressed in EOC tissues and cell lines, stable GINS2 knockdown in SKOV-3 cells could significantly inhibit cell proliferation and induce cell cycle arrest and cell apoptosis [40]. In current study, survival analysis of the hub genes related to target genes of miR-182 showed that GINS2 was highly associated with poor prognosis of patients with OC.

In current study, we identified that novel candidate target genes for miR-182 were involved in the regulation of crucial biological processes in OC, such as DCN, AKT3, and TIMP2. DCN is a component of connective tissue, binds to type I collagen fibrils and plays a role in collagen fibrillogenesis when helps to orient fibers. Some previous studies showed that DCN played an important function in cancer development and metastasis. For example, Ehlen et al. [41] found that DCN gene was up-regulated in OC cells and played an important role in SEOC angiogenesis and tumor progression; highly expressed DCN could increase angiogenesis and tumor cell invasiveness in bladder cancer [42]. Our current study revealed that DCN mRNA was decreased in OC compared with NOTs, which was consistent with some previous studies [43–46], we hypothesized that DCN can act as a tumor suppressor in OC. AKT3 is a homologous gene that belongs to the serine**/**threonine protein kinase AKT subfamily and is critical in the AKT signal pathway [41, 47]. The expression and activation of AKT3 mediates cancer progression and controls cellular processes such as cell growth, proliferation, apoptosis, and invasion [48]. Some studies found that AKT3 was up-regulated in many types of cancers and knockdown of AKT3 isoforms could abrogate the growth of tumors through inducing cell apoptosis and inhibiting proliferation [49–51]. Moreover, lots of evidence suggested that AKT3 was regulated by miRNAs, such as miR-497 [52], miR-338 [53], and miR-16 [54], which could suppress cancer progression. In line with these findings, we hypothesized that AKT3 can be identified as a target gene of miR-182 in OC. TIMP2 is one of four well-known members of the TIMP family: TIMP1, TIMP2, TIMP3, and TIMP4. The function of TIMP2 in carcinogenesis is multifaceted. For example, some previous studies showed that an increasing level of TIMP2 can promote cellular proliferation and invasion in some tumors [55, 56]. On the contrary, some studies found that TIMP2 could inhibit vascular endothelial growth factor A (VEGF-A)-induced endothelial cell proliferation and angiogenesis by binding to α3β1 integrin [57]. Moreover, TIMP2 could prevent the activation of tyrosine kinase receptors in tumor cells, including focal adhesion kinase [58], AKT [59] and epithelial growth factor receptor [60], which played key roles in tumor migration and growth. Our current study revealed that TIMP2 mRNA was decreased in OC compared with NOTs, which was consistent with some previous studies [61–63]. To the best of our knowledge, few studies have shown that the down-regulation pattern of TIMP2 occurs in OC tissues. Thus, further investigation is required to explore the ectopic expression of TIMP2 in OC.

## Conclusions

In summary, the results presented here suggest that miR-182 plays an important role in the biology of OC. However, further studies in vitro and in vivo are still needed on the pathogenesis to validate the role of miR-182-regulated molecular networks in OC.

## Supplementary information

**Additional file 1: Table S1.** Node-degree analysis of the 28 hub genes (Degree ≥10).

**Additional file 2: Figure S1.** The correlated expression of gene and hsa-miR-182-5p (miR-182) in patients with ovarian cancer. a: TPM1, b: COL1A1, c: PDGFRA, d: UBE2B, e: MEF2C, f: SNAI2, g: CACNA2D1, h: RECK, i: FOXO1, j: FBN1, k: ANTXR2, l: NKX3–1, m: TIMP2, n: AKT3, o: RBPMS, p: EGLN3, q: DERL1, r: PRKD1, s: SLC2A13, t: MAF, u: DCN.

## Data Availability

The datasets used and/or analysed during the current study are available from the corresponding author on reasonable request.
