# Supplementary material for: Prognostic values and prospective pathway signaling of MicroRNA-182 in ovarian cancer: a study based on gene expression omnibus (GEO) and bioinformatics analysis
Source: J Ovarian Res. 2019 Nov 8;12:106. doi: 10.1186/s13048-019-0580-7 (PMC6839211; doi:10.1186/s13048-019-0580-7)
Supplement: Supplementary file 1 — Additional file 1: Table S1. Node-degree analysis of the 28 hub genes (Degree ≥10). [file 13048_2019_580_MOESM1_ESM.doc]

| a TPM1 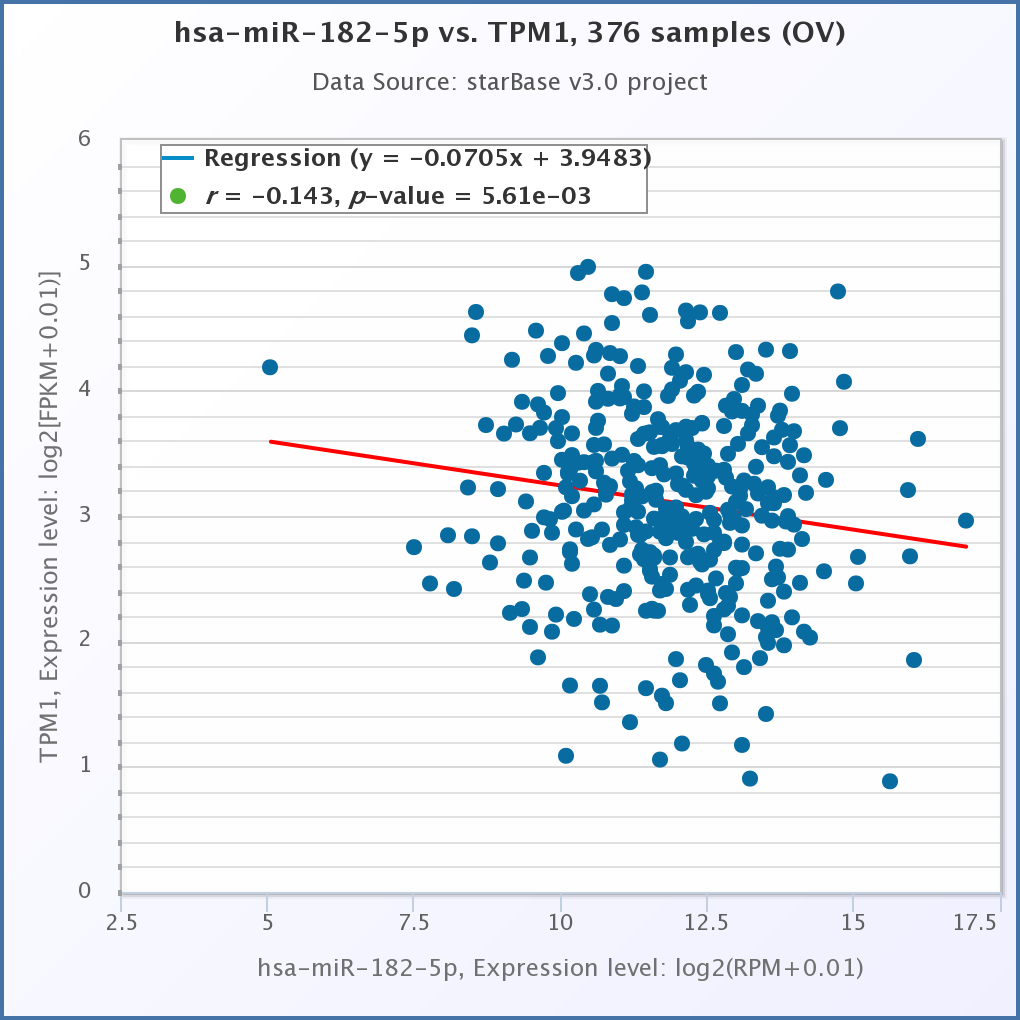 | b COL1A1  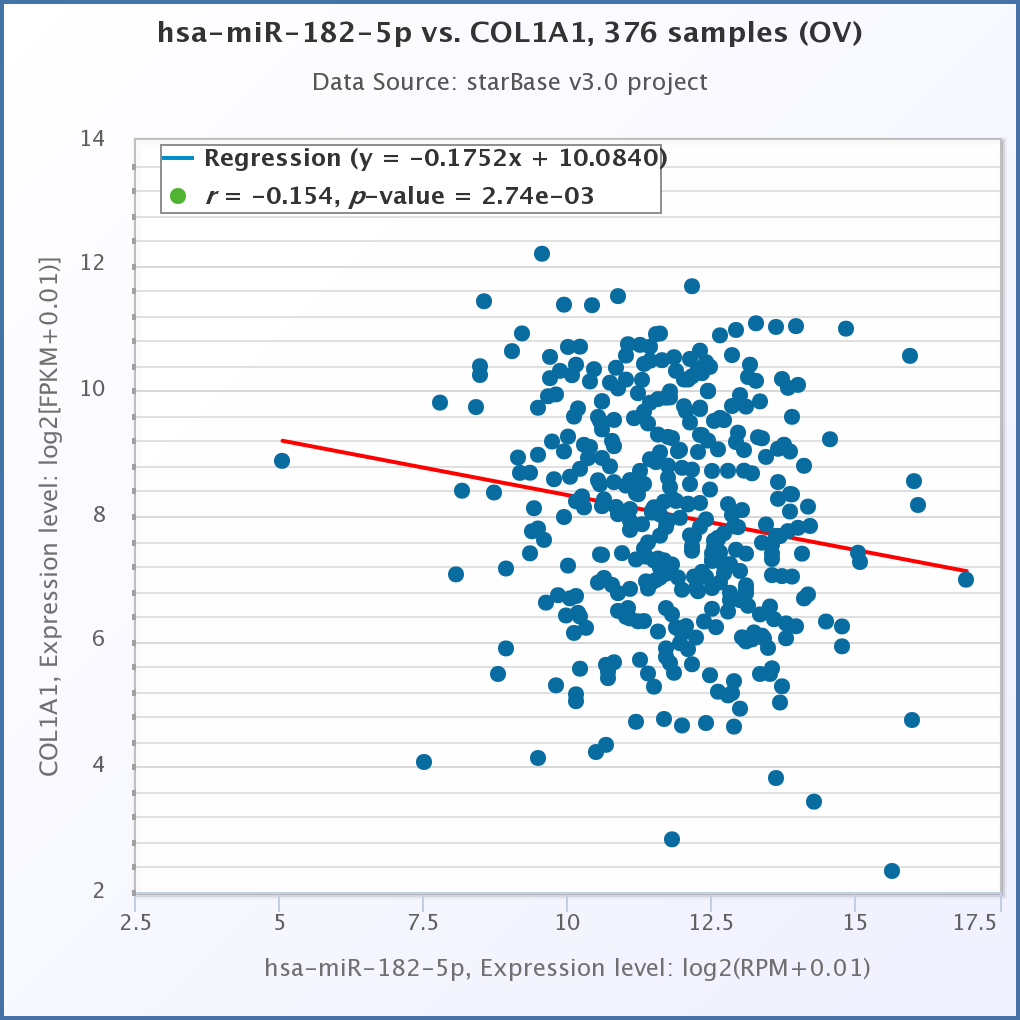 | c PDGFRA  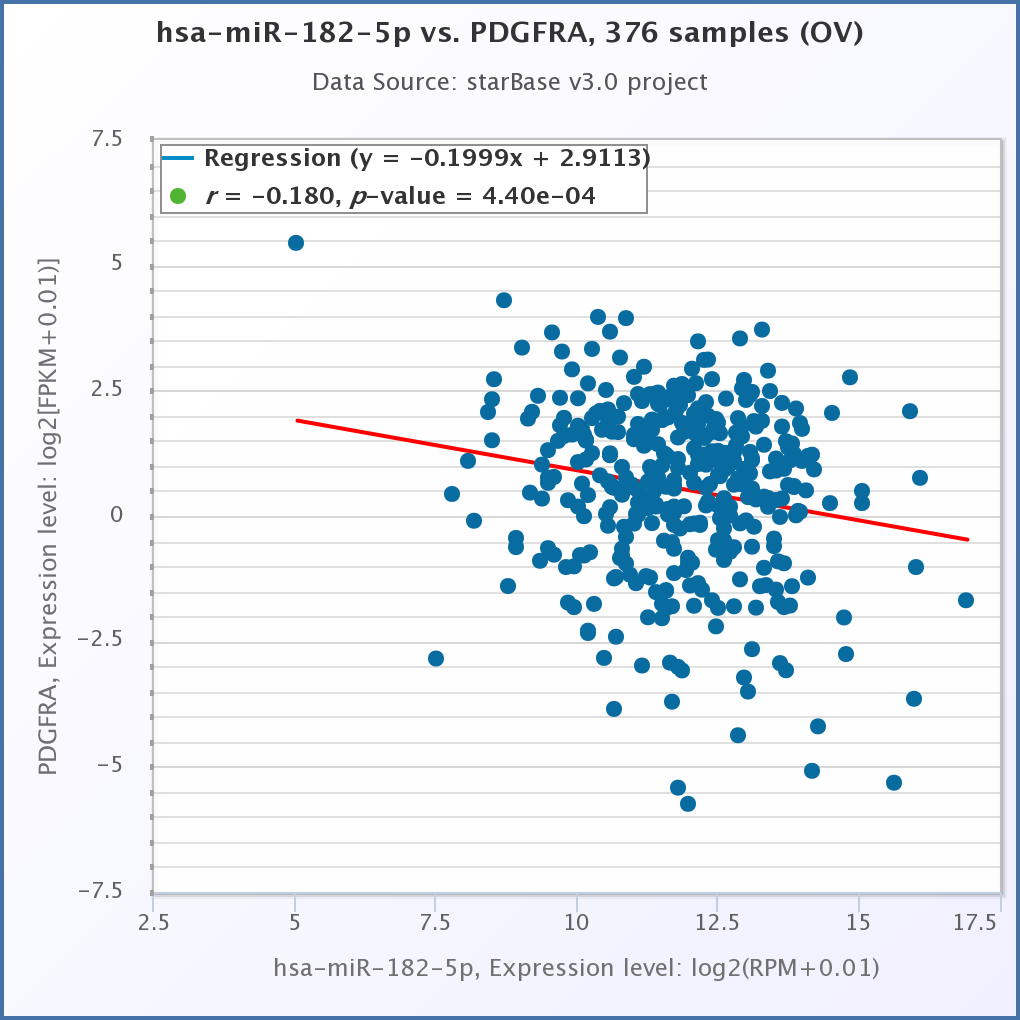 |
| --- | --- | --- |
| d UBE2B  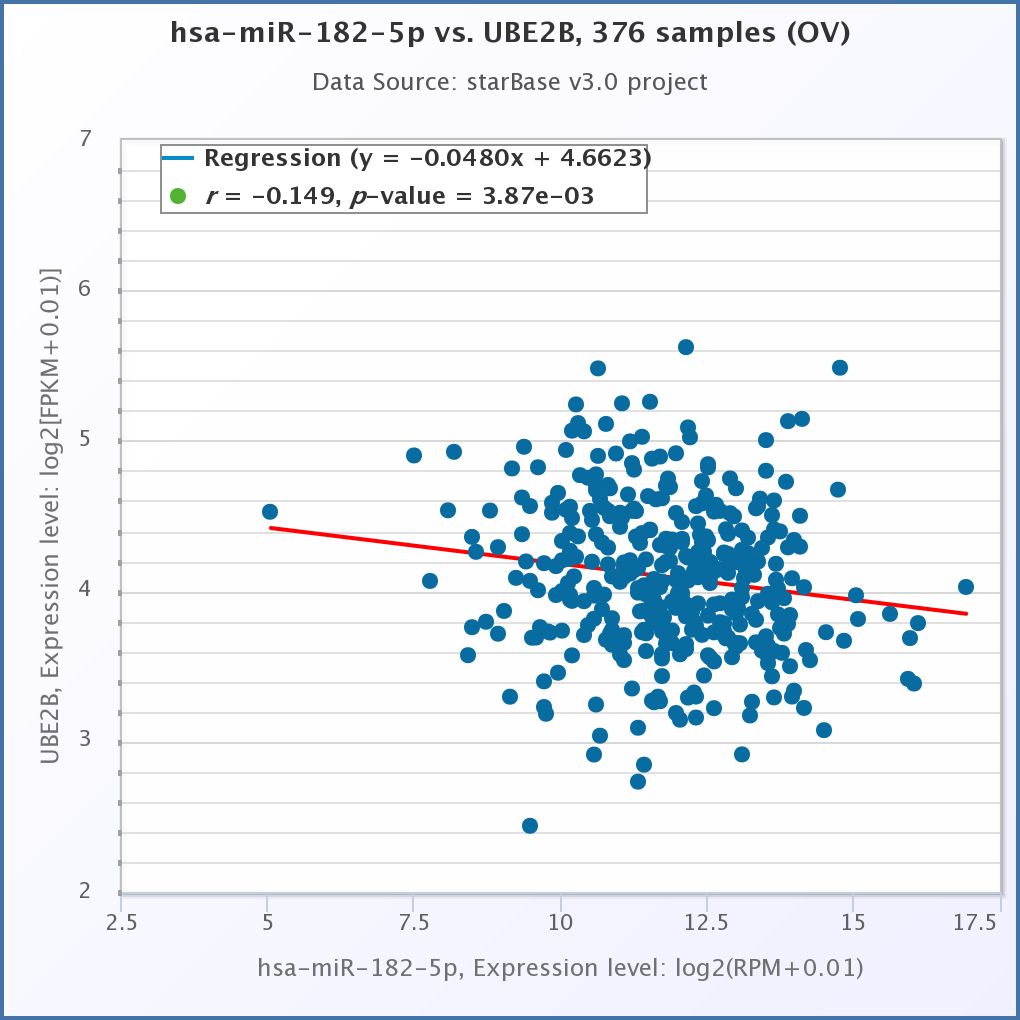 | e MEF2C  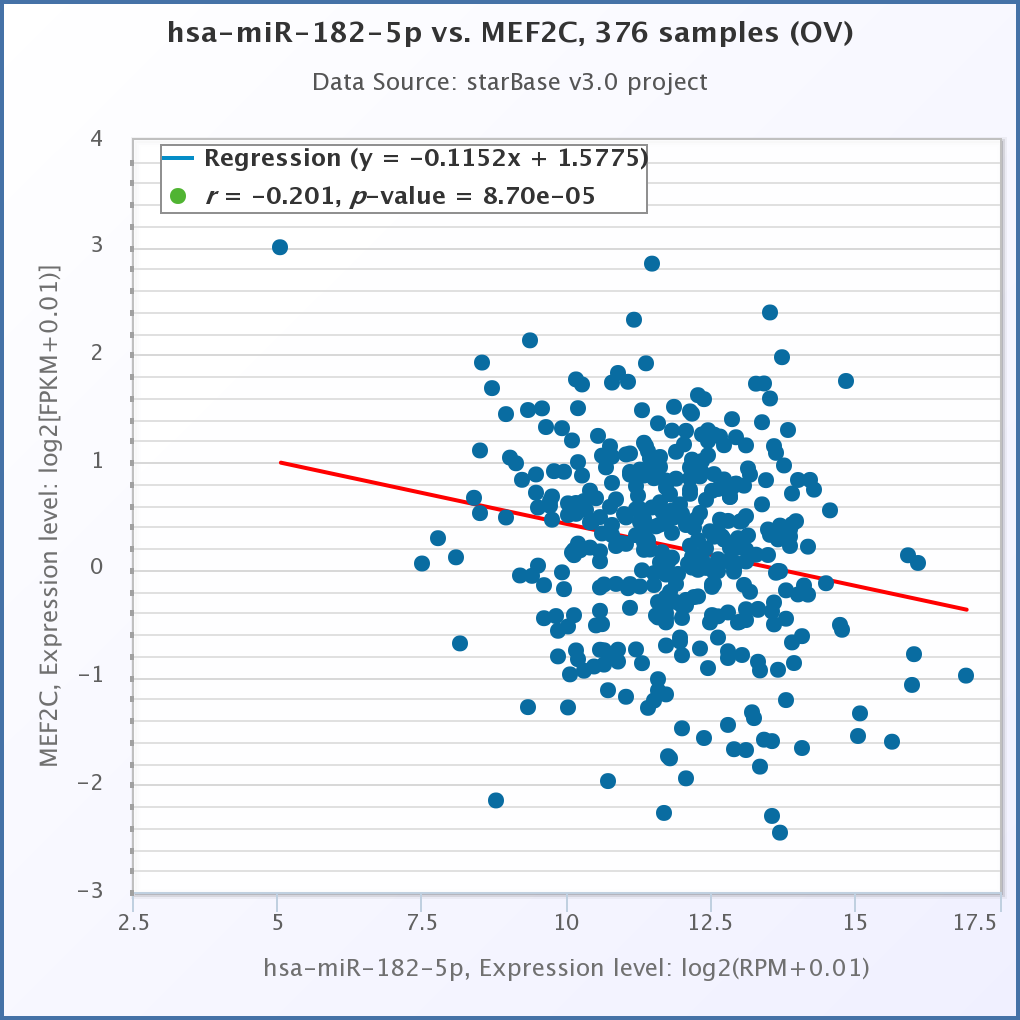 | f SNAI2  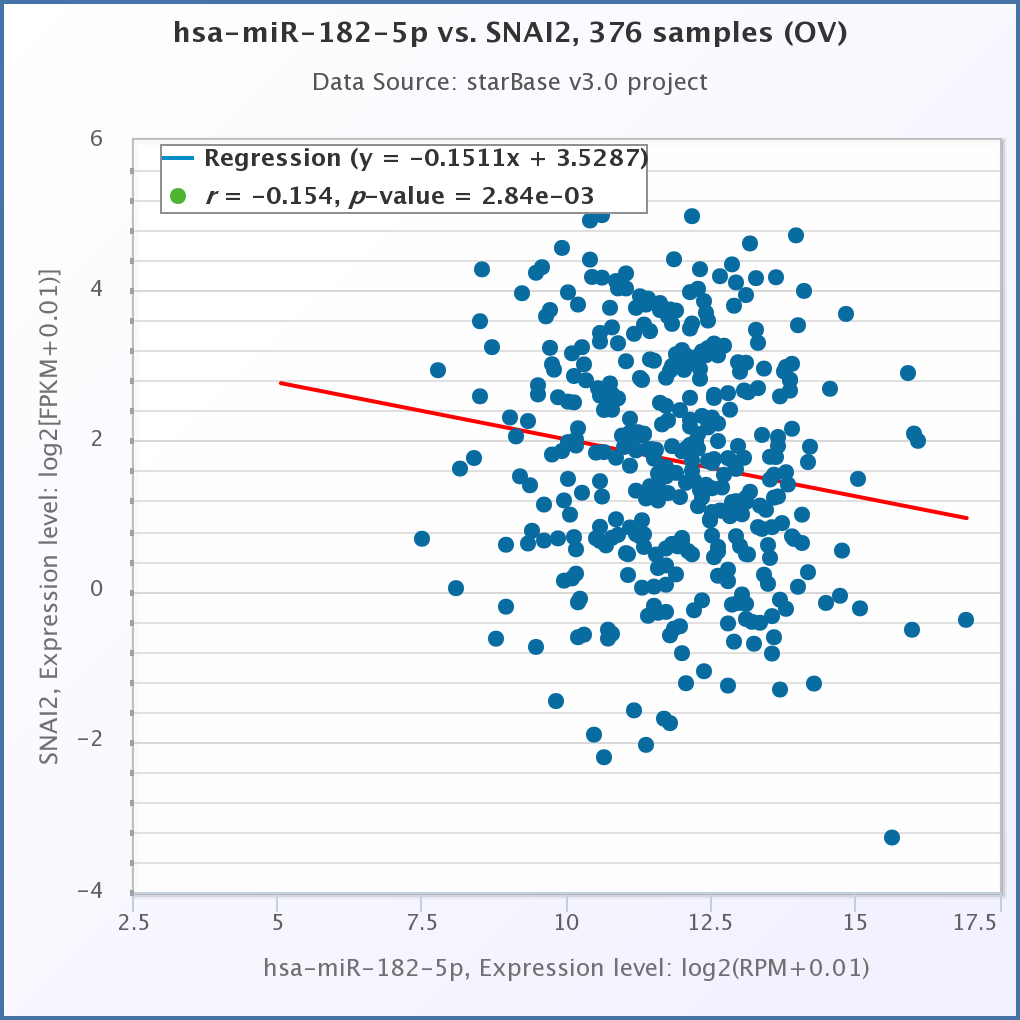 |
| g CACNA2D1  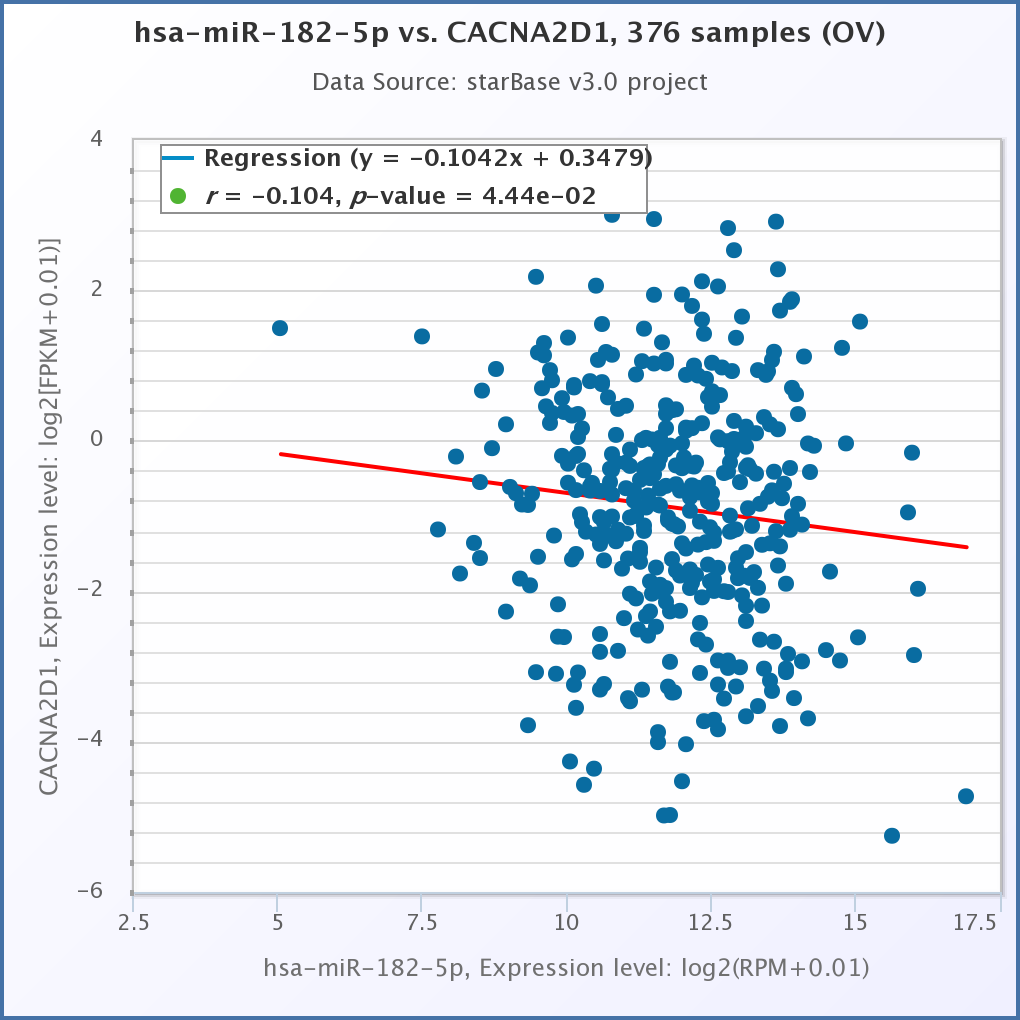 | h RECK  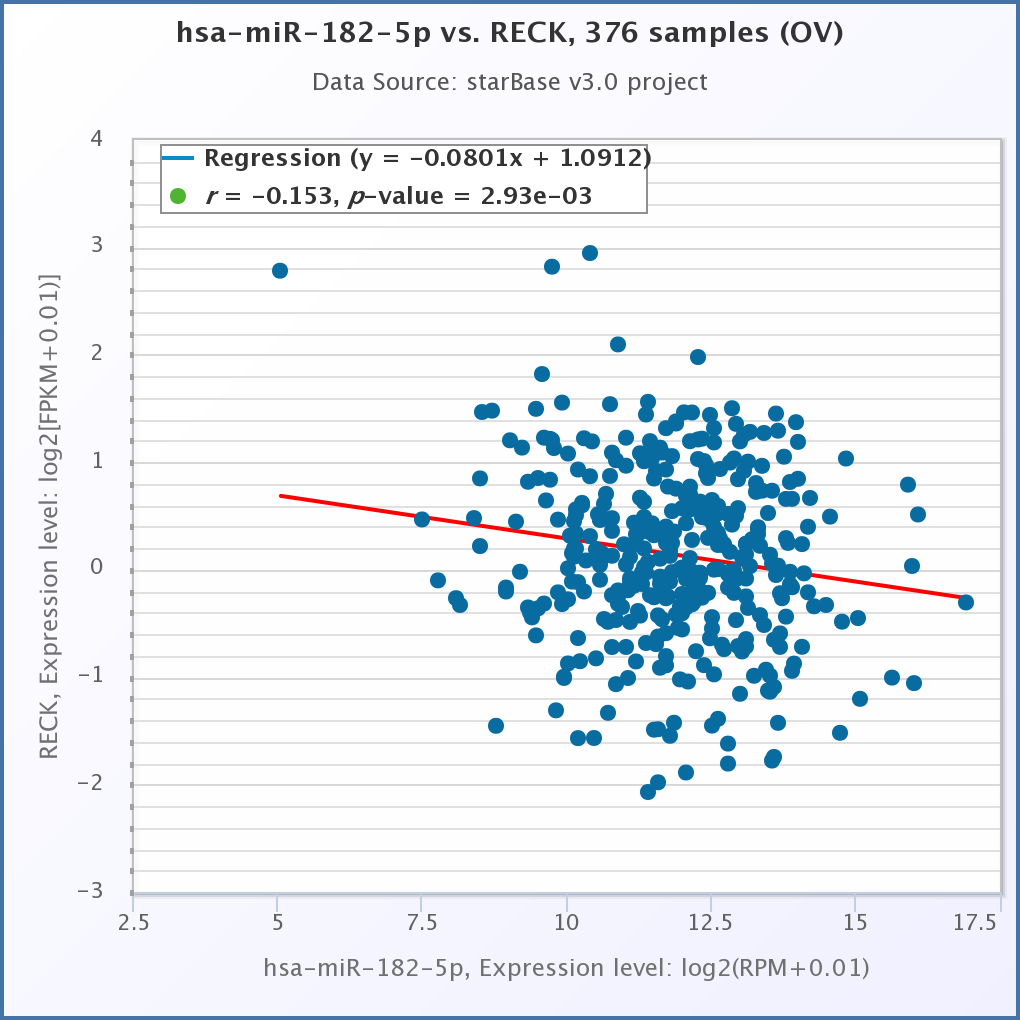 | i FOXO1  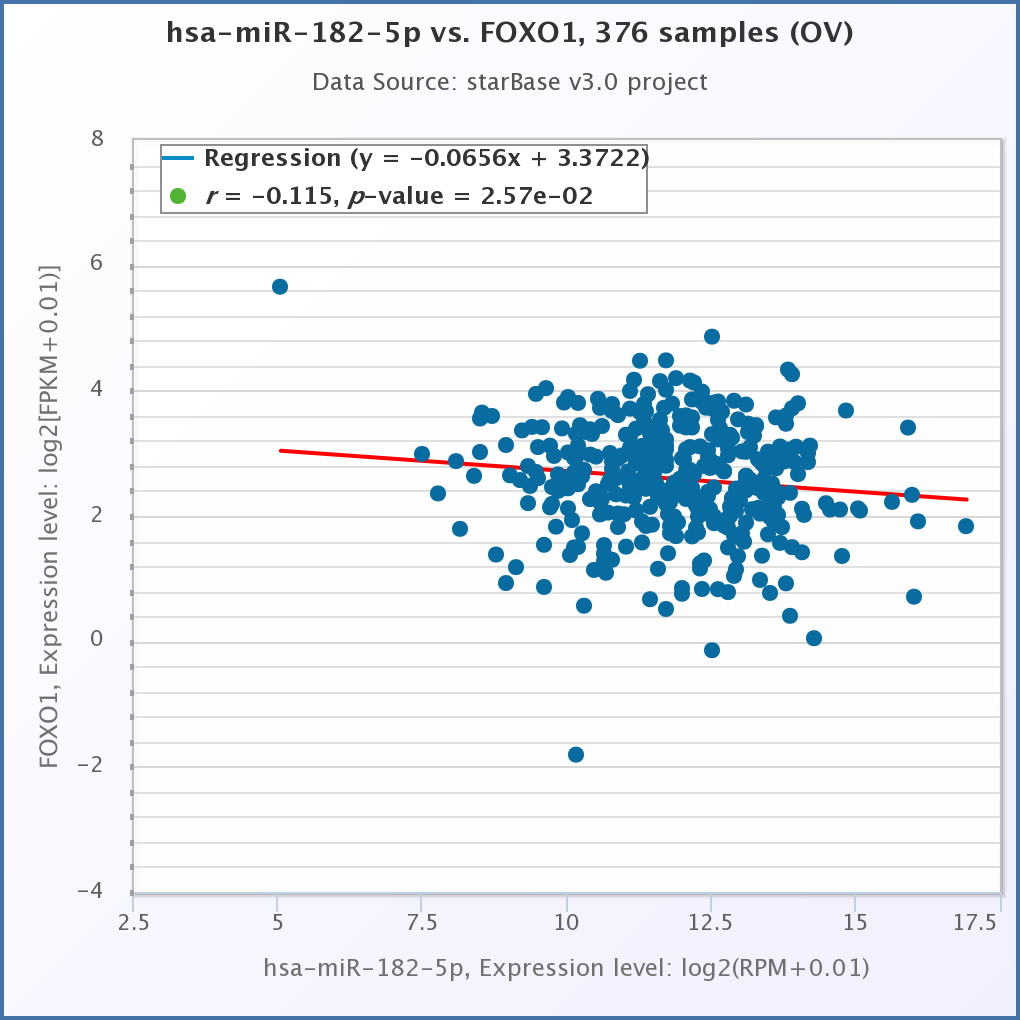 |
| j FBN1  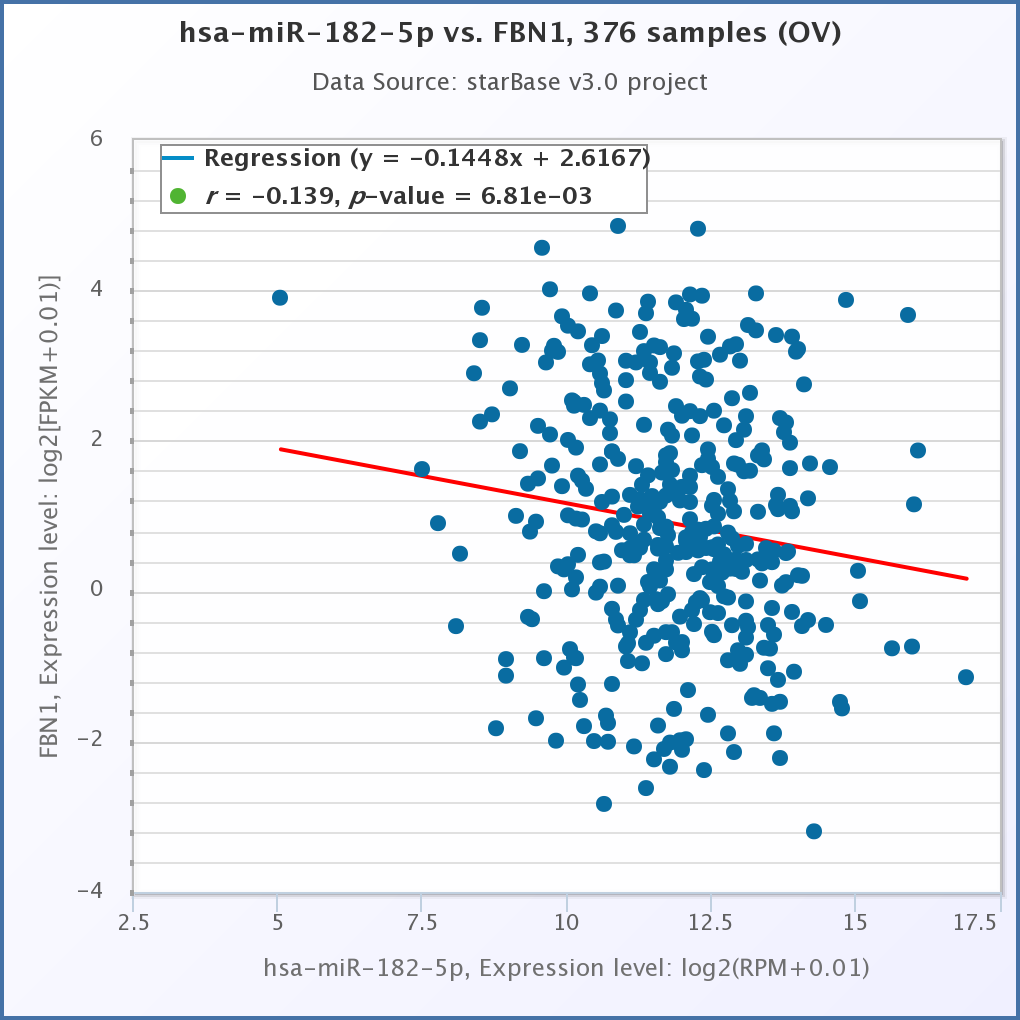 | k ANTXR2  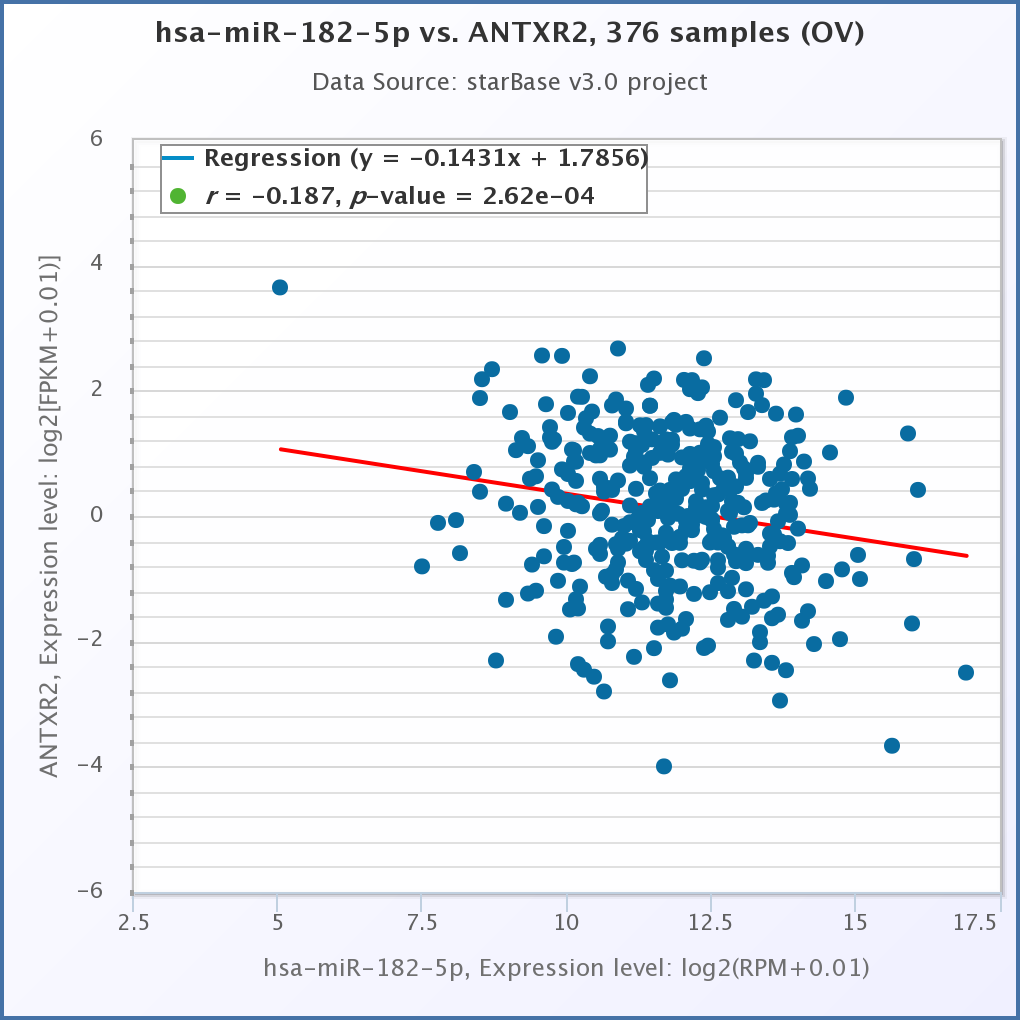 | l NKX3-1  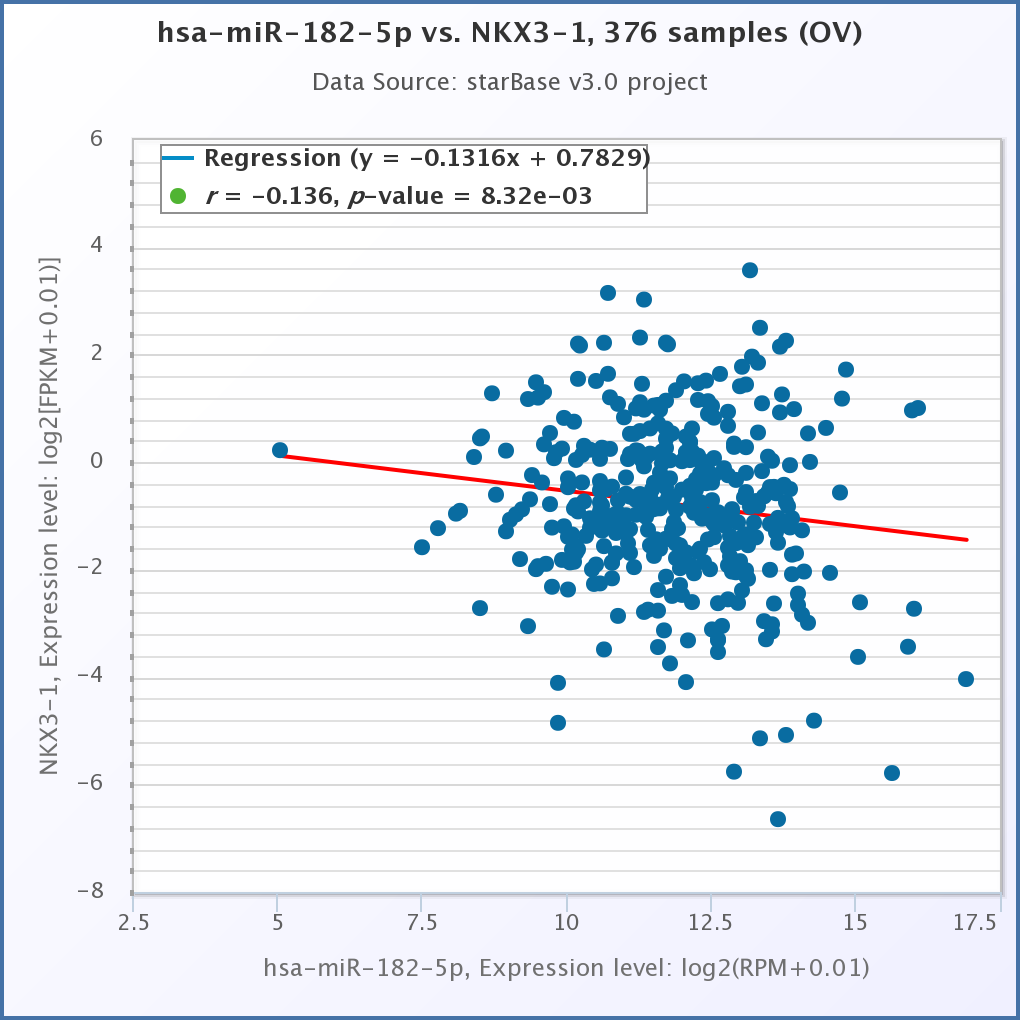 |
| m TIMP2  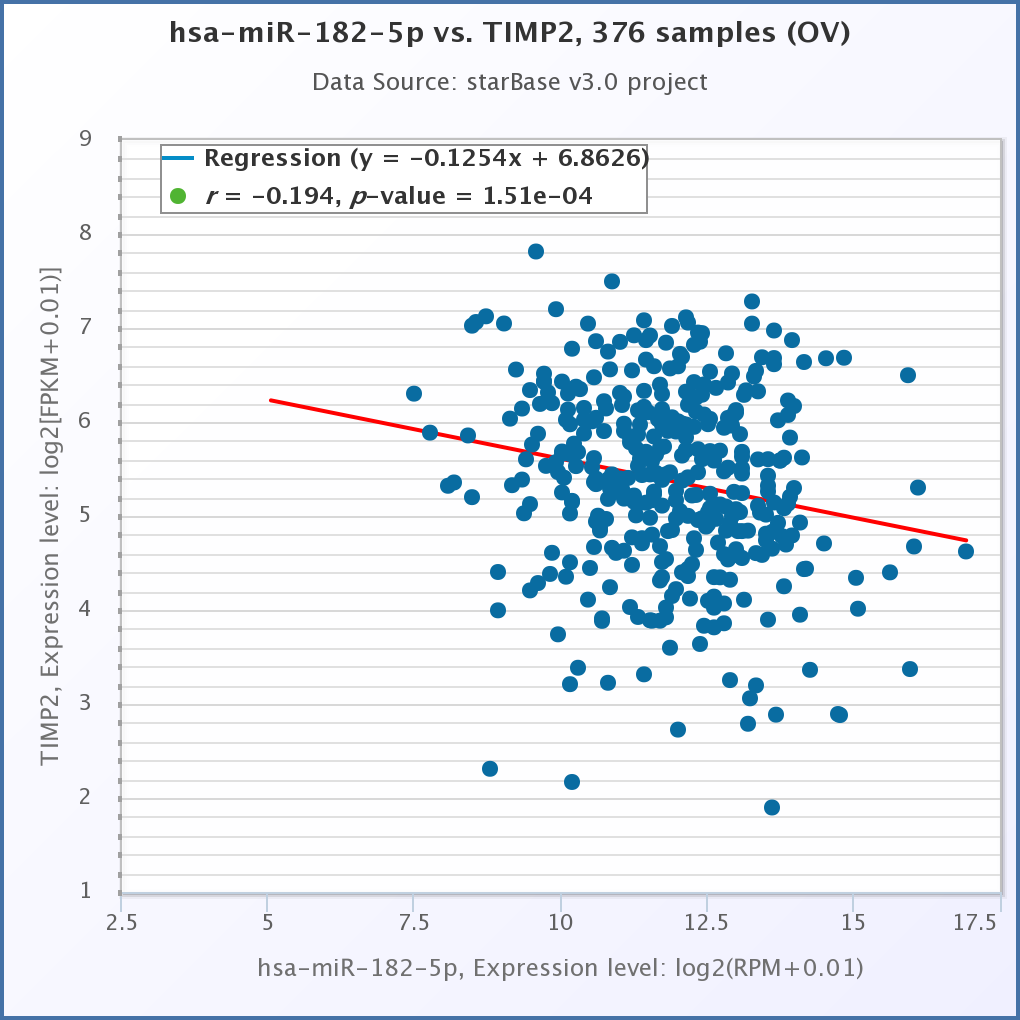 | n AKT3  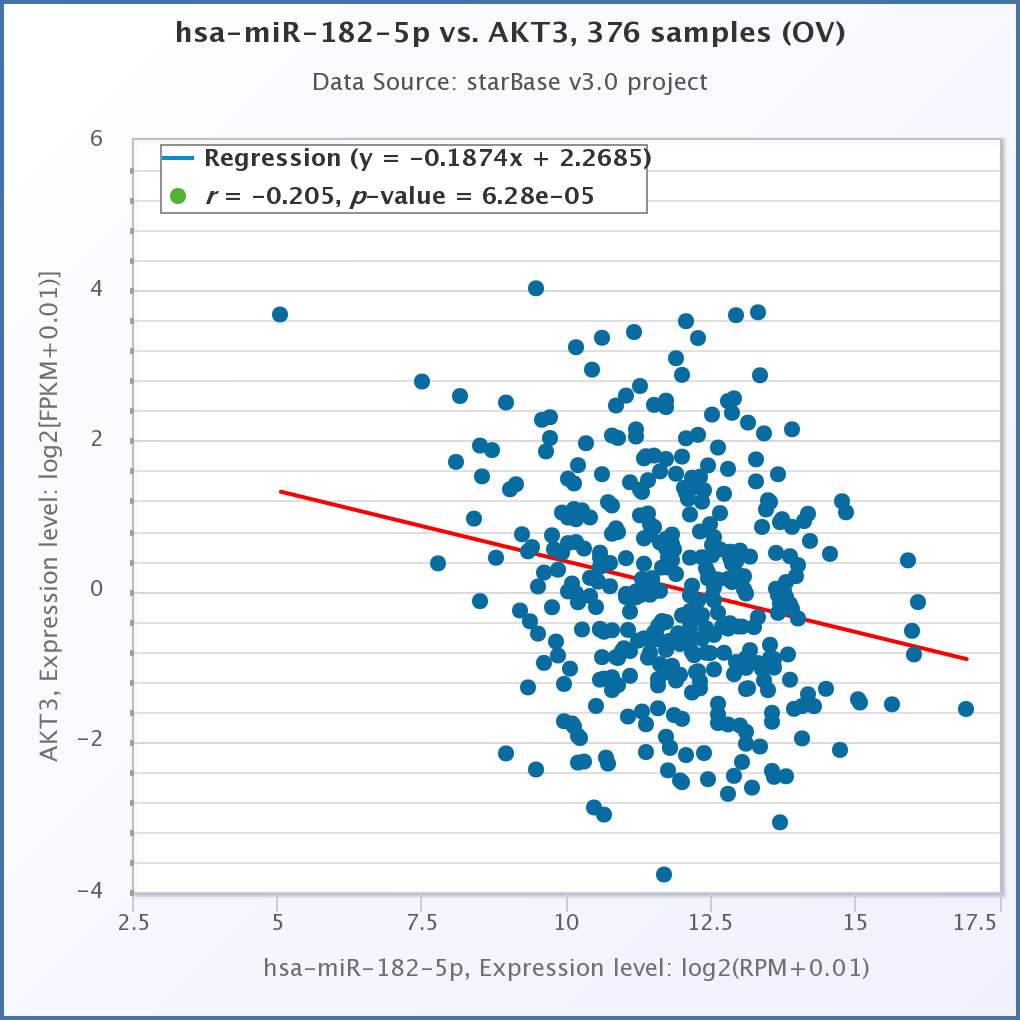 | o RBPMS  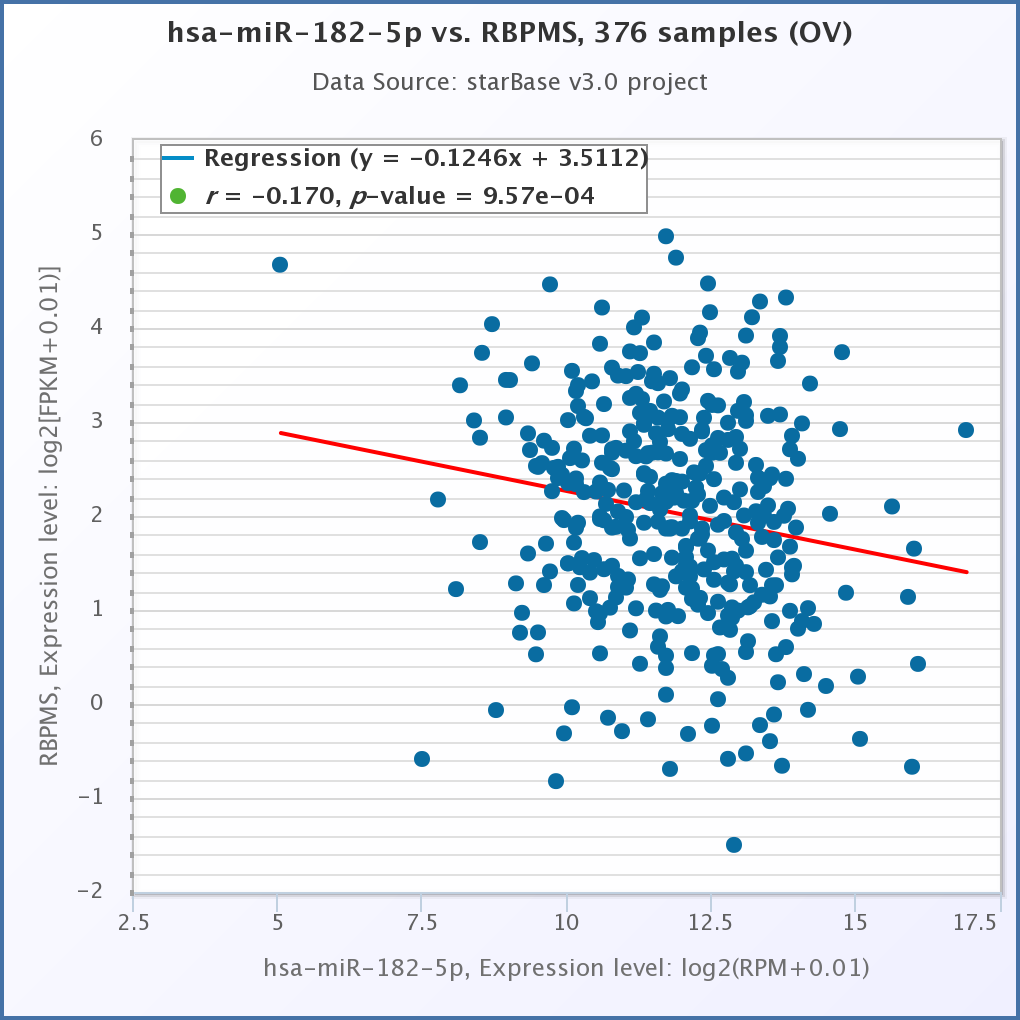 |
| p EGLN3  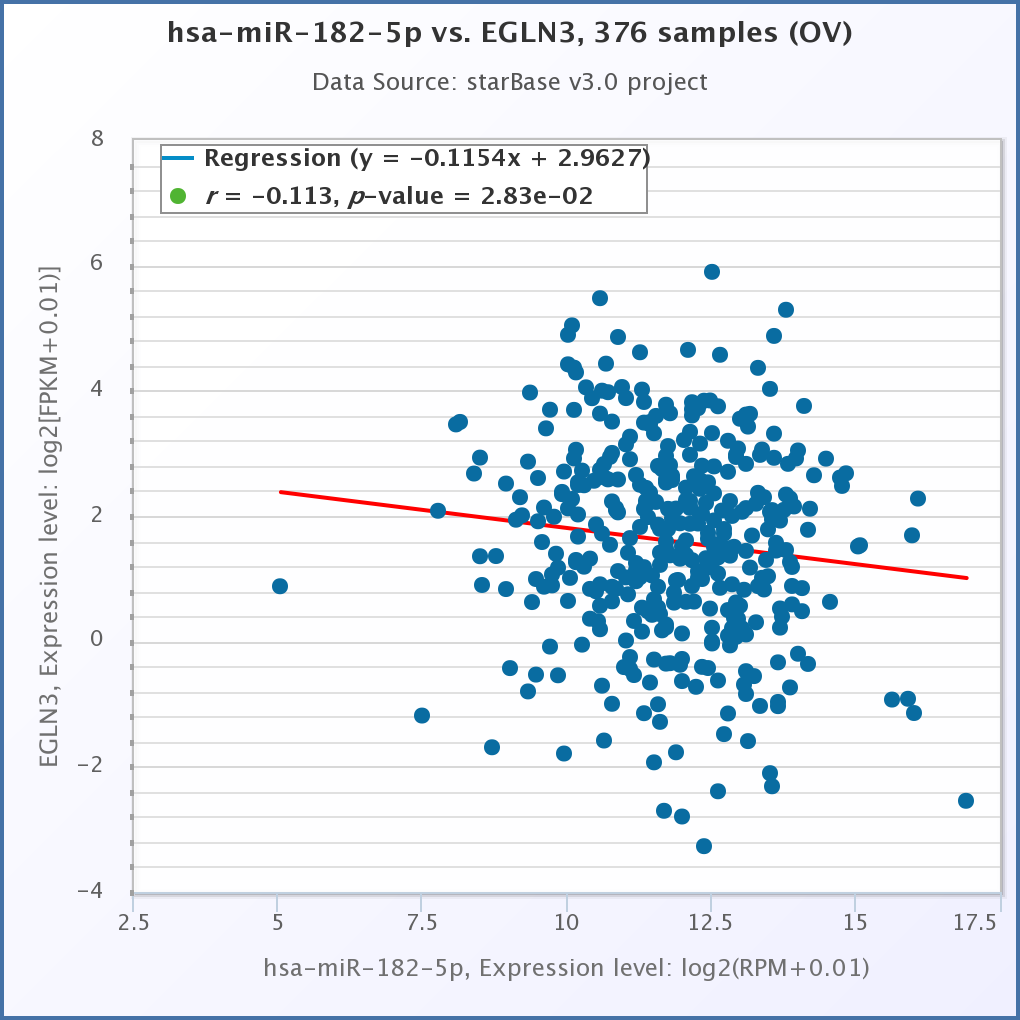 | q DERL1  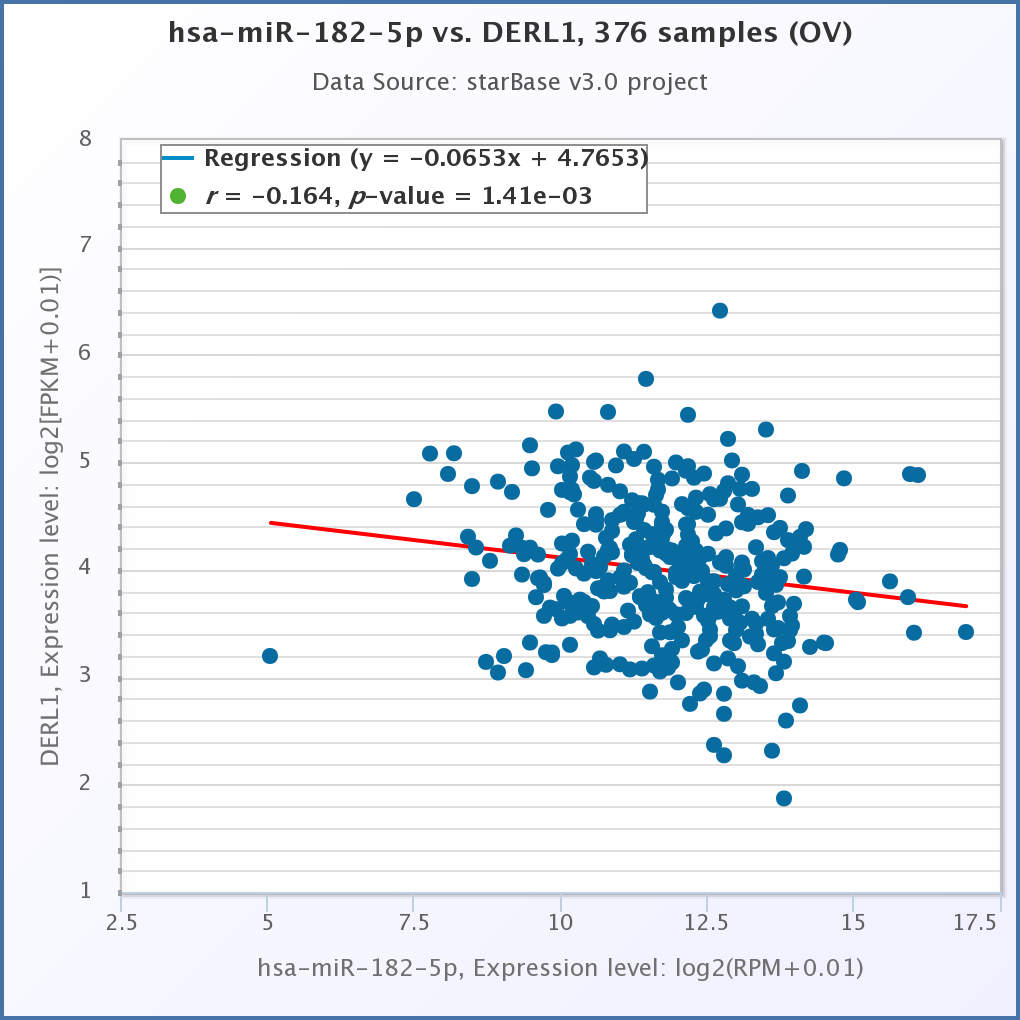 | r PRKD1  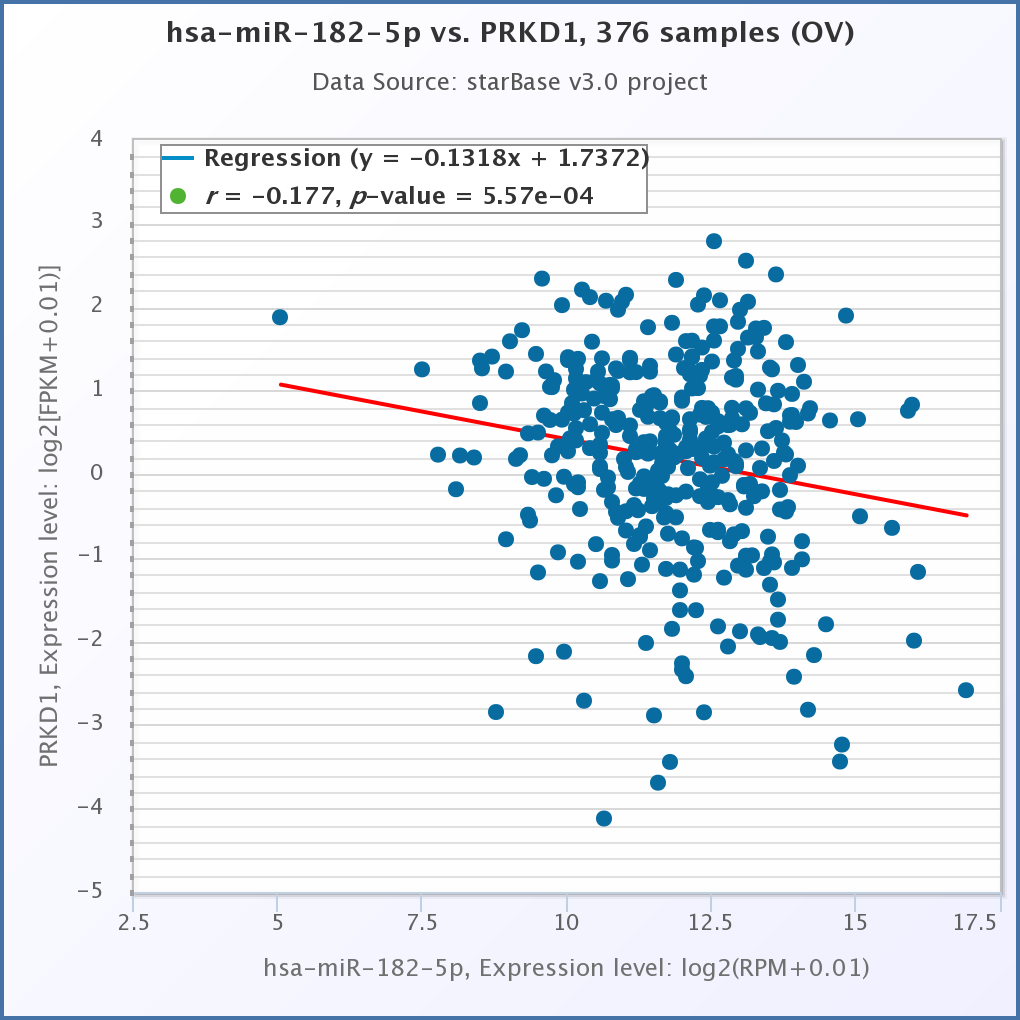 |
| s SLC2A13  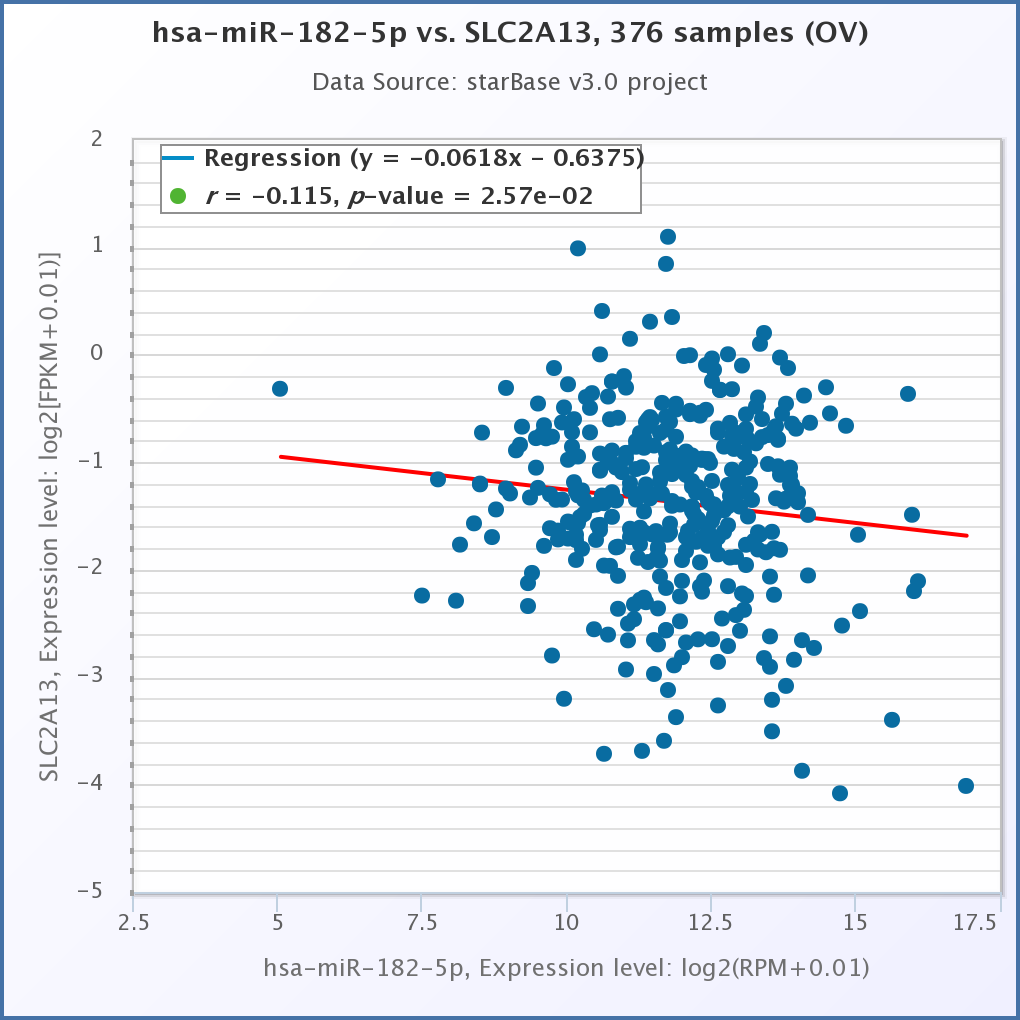 | t MAF  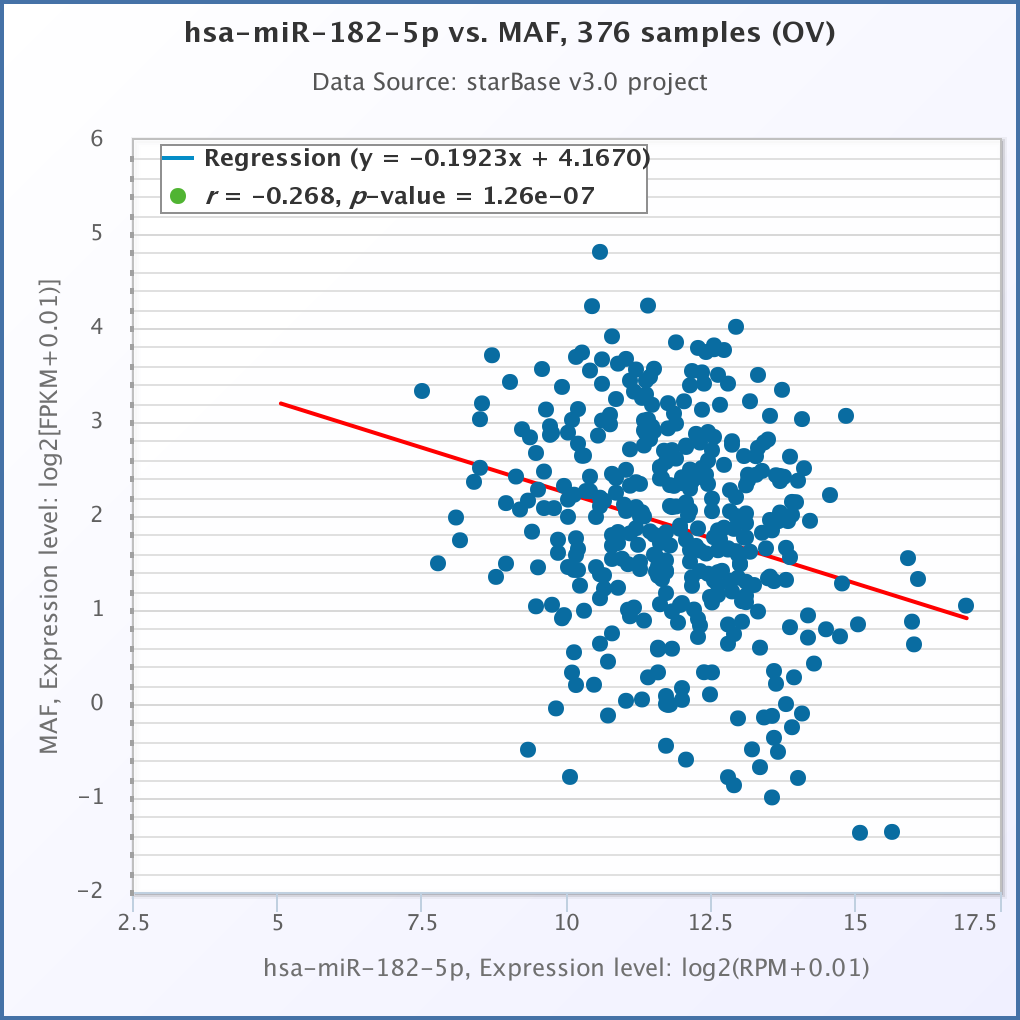 | u DCN  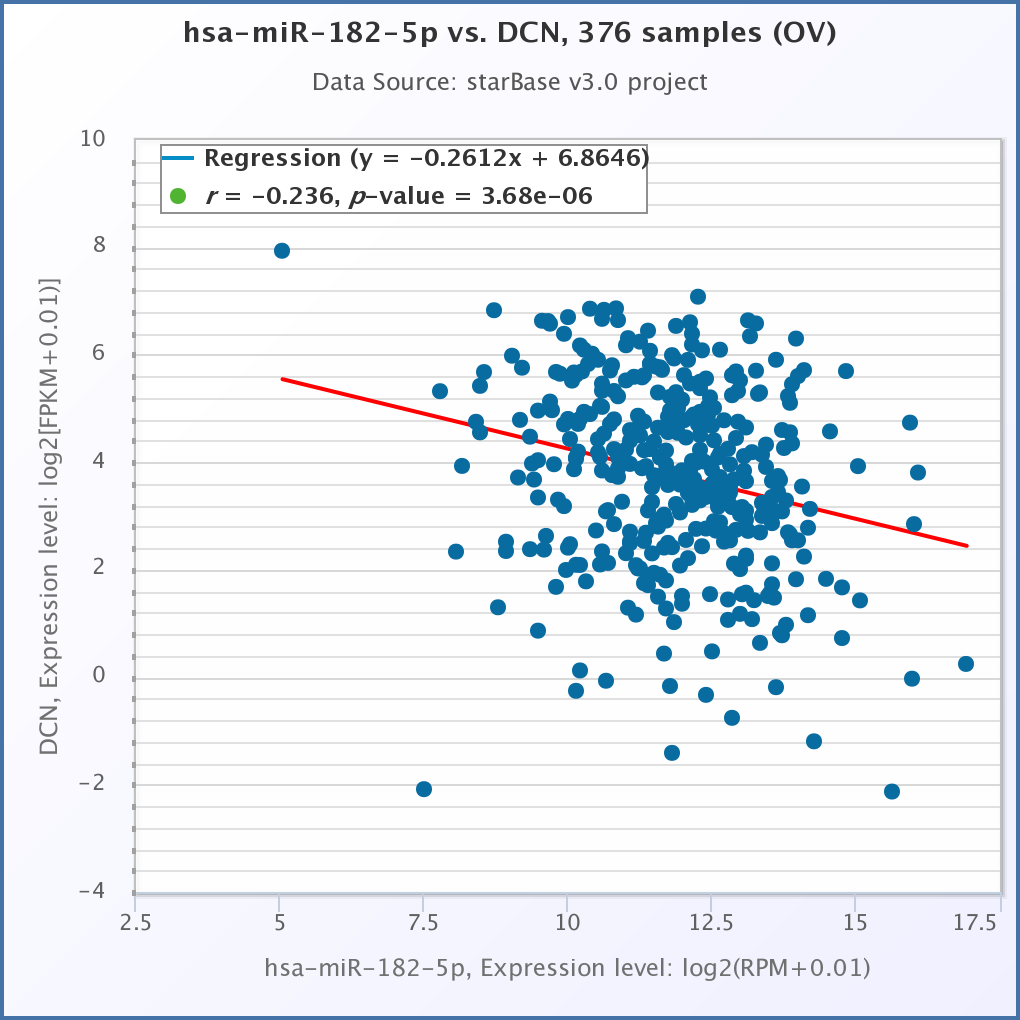 |

Figure. S1 The correlated expression of gene and hsa-miR-182-5p (miR-182) in patients with ovarian cancer. a: TPM1, b: COL1A1, c: PDGFRA, d: UBE2B, e: MEF2C, f: SNAI2, g: CACNA2D1, h: RECK, i: FOXO1, j: FBN1, k: ANTXR2, l: NKX3-1, m: TIMP2, n: AKT3, o: RBPMS, p: EGLN3, q: DERL1, r: PRKD1, s: SLC2A13, t: MAF, u: DCN
