# Supplementary material for: Prognostic values and prospective pathway signaling of MicroRNA-182 in ovarian cancer: a study based on gene expression omnibus (GEO) and bioinformatics analysis
Source: J Ovarian Res. 2019 Nov 8;12:106. doi: 10.1186/s13048-019-0580-7 (PMC6839211; doi:10.1186/s13048-019-0580-7)
Supplement: Supplementary file 2 — Additional file 2: Figure S1. The correlated expression of gene and hsa-miR-182-5p (miR-182) in patients with ovarian cancer. a: TPM1, b: COL1A1, c: PDGFRA, d: UBE2B, e: MEF2C, f: SNAI2, g: CACNA2D1, h: RECK, i: FOXO1, j: FBN1, k: ANTXR2, l: NKX3–1, m: TIMP2, n: AKT3, o: RBPMS, p: EGLN3, q: DERL1, r: PRKD1, s: SLC2A13, t: MAF, u: DCN. [file 13048_2019_580_MOESM2_ESM.doc]

Table. S1 Node-degree analysis of the 28 hub genes (Degree ≥ 10).

| Node_name | Degree |
| --- | --- |
| CDC45 | 37 |
| MCM2 | 37 |
| MCM4 | 36 |
| CDC6 | 35 |
| MCM3 | 34 |
| MCM7 | 34 |
| MCM10 | 34 |
| MCM5 | 34 |
| MCM6 | 31 |
| NCAPG | 29 |
| GINS2 | 27 |
| CDT1 | 26 |
| ORC1 | 25 |
| TOP2A | 25 |
| SMC4 | 23 |
| CDC7 | 23 |
| KIF23 | 23 |
| RPA3 | 23 |
| SMC2 | 23 |
| DTL | 23 |
| BIRC5 | 22 |
| PRC1 | 22 |
| NCAPH | 21 |
| ORC4 | 20 |
| ORC5 | 20 |
| GINS4 | 20 |
| ORC3 | 19 |
| RACGAP1 | 19 |
| RAD51 | 19 |
| KIF2C | 19 |
| ORC2 | 18 |
| ORC6 | 17 |
| GINS3 | 15 |
| HMMR | 15 |
| NCAPD2 | 14 |
| GINS1 | 14 |
| CDK4 | 14 |
| MCMBP | 11 |
